# Identification of Host Cytosolic Sensors and Bacterial Factors Regulating the Type I Interferon Response to *Legionella pneumophila*

**DOI:** 10.1371/journal.ppat.1000665

**Published:** 2009-11-20

**Authors:** Kathryn M. Monroe, Sarah M. McWhirter, Russell E. Vance

**Affiliations:** Division of Immunology and Pathogenesis, Department of Molecular and Cell Biology, University of California, Berkeley, California, United States of America; Tufts University School of Medicine, United States of America

## Abstract

*Legionella pneumophila* is a gram-negative bacterial pathogen that replicates in host macrophages and causes a severe pneumonia called Legionnaires' Disease. The innate immune response to *L. pneumophila* remains poorly understood. Here we focused on identifying host and bacterial factors involved in the production of type I interferons (IFN) in response to *L. pneumophila*. It was previously suggested that the delivery of *L. pneumophila* DNA to the host cell cytosol is the primary signal that induces the type I IFN response. However, our data are not easily reconciled with this model. We provide genetic evidence that two RNA-sensing proteins, RIG-I and MDA5, participate in the IFN response to *L. pneumophila*. Importantly, these sensors do not seem to be required for the IFN response to *L. pneumophila* DNA, whereas we found that RIG-I was required for the response to *L. pneumophila* RNA. Thus, we hypothesize that bacterial RNA, or perhaps an induced host RNA, is the primary stimulus inducing the IFN response to *L. pneumophila*. Our study also identified a secreted effector protein, SdhA, as a key suppressor of the IFN response to *L. pneumophila*. Although viral suppressors of cytosolic RNA-sensing pathways have been previously identified, analogous bacterial factors have not been described. Thus, our results provide new insights into the molecular mechanisms by which an intracellular bacterial pathogen activates and also represses innate immune responses.

## Author Summary

Initial detection of invading microorganisms is one of the primary tasks of the innate immune system. However, the molecular mechanisms by which pathogens are recognized remain incompletely understood. Here, we provide evidence that an immunosurveillance pathway (called the RIG-I/MDA5 pathway), thought primarily to detect viruses, is also involved in the innate immune response to an intracellular bacterial pathogen, *Legionella pneumophila*. In the response to viruses, the RIG-I/MDA5 immunosurveillance pathway has been shown to respond to viral RNA or DNA. We found that the RIG-I pathway was required for the response to *L. pneumophila* RNA, but was not required for the response to *L. pneumophila* DNA. Thus, one explanation of our results is that *L. pneumophila* RNA may access the host cell cytosol, where it triggers the RIG-I/MDA5 pathway. This is unexpected since bacteria have not previously been thought to translocate RNA into host cells. We also found that *L. pneumophila* encodes a secreted bacterial protein, SdhA, which suppresses the RIG-I/MDA5 pathway. Several viral repressors of the RIG-I/MDA5 pathway have been described, but bacterial repressors of RIG-I/MDA5 are not known. Thus, our study provides novel insights into the molecular mechanisms by which the immune system detects bacterial infection, and conversely, by which bacteria suppress innate immune responses.

## Introduction

The intracellular bacterium *Legionella pneumophila* has become a valuable model for the study of immunosurveillance pathways. *L. pneumophila* is a motile gram-negative bacterium that is the cause of a severe pneumonia called Legionnaires' Disease [1]. In the environment, *L. pneumophila* is believed to replicate in various species of freshwater amoebae. In humans, *L. pneumophila* causes disease by replicating within alveolar macrophages in the lung [2]. Replication in macrophages and amoebae requires a type IV secretion system that the bacterium uses to inject effector proteins into the host cell cytosol [3]. These effectors are believed to orchestrate the creation of an intracellular vacuole in which *L. pneumophila* can replicate. Interestingly, there appears to be considerable redundancy among the effectors, and there are few examples of single effector mutations that have a large effect on intracellular replication of *L. pneumophila*. One *L. pneumophila* effector required for intracellular replication is SdhA [4], but the mechanism by which SdhA acts on host cells remains uncertain [4].

A variety of immunosurveillance pathways that detect *L. pneumophila* infection have been described [5],[6],[7],[8]. The best characterized cytosolic immunosurveillance pathway requires the host proteins Naip5 and Ipaf to detect the cytosolic presence of *L. pneumophila* flagellin, leading to activation of caspase-1, rapid pyroptotic macrophage death, and efficient restriction of bacterial replication [9],[10],[11],[12],[13]. *L. pneumophila* has also been observed to induce transcriptional activation of type I interferon (IFN) genes in macrophages and epithelial-like cell lines by a mechanism that remains incompletely characterized [14],[15]. Induction of type I IFNs by *L. pneumophila* is independent of the flagellin-sensing pathway [16], but also appears to contribute to restriction of bacterial replication in macrophages [16],[17] and epithelial-like cell lines [14].

Type I IFNs are an important class of cytokines that orchestrate diverse immune responses to pathogens [18]. Encoded by a single IFNβ gene as well as multiple IFNα and other (e.g., IFNε, κ, δ, ζ) genes, type I IFNs are transcriptionally induced by a number of immunosurveillance pathways, including Toll-like receptors (TLRs) and a variety of cytosolic sensors [19]. For example, cytosolic RNA is recognized by two distinct helicase and CARD-containing sensors, RIG-I and MDA5 [20], that signal through the adaptor IPS-1 (also called MAVS, CARDIF, or VISA) [21],[22],[23],[24],[25]. The cytosolic presence of DNA also induces type I IFNs, but this phenomenon is less well understood [15],[26]. Studies with *Ips-1*-deficient mice have indicated that cytosolic DNA can signal independently of *Ips-1* in many cell types, including macrophages [25]. However, cytosolic responses to DNA appear to require IPS-1 in certain cell types, including 293T cells [26],[27]. Indeed, two recent reports have described a pathway by which AT-rich DNA can signal via IPS-1 [28],[29]. In this pathway, DNA is transcribed by RNA polymerase III to form an RNA intermediate that can be sensed by RIG-I. The RNA Pol III pathway appears to be operational in macrophages, but is redundant with other DNA-sensing pathways in these cells. A couple of reports have proposed that DAI (also called ZBP-1) is a cytosolic DNA-sensor [30],[31], but *Zbp1*-deficient mice appear to respond normally to cytosolic DNA [32], consistent with the existence of multiple cytosolic sensors for DNA. Other small molecule compounds, such as cyclic-di-GMP and DMXAA, can also trigger cytosolic immunosurveillance pathways leading to induction of type I IFNs, but these remain to be fully characterized [33],[34],[35].

Type I IFNs are typically considered antiviral cytokines that act locally to induce an antiviral state and systemically to induce cellular innate and adaptive immune responses [19]. Mice deficient in the type I IFN receptor (*Ifnar*) are unable to respond to type I IFNs, and are highly susceptible to viral infections. Interestingly, most bacterial infections also trigger production of type I IFNs, but the physiological significance of type I IFNs in immune defense against bacteria is complex. Type I IFN appears to protect against infection with group B *Streptococcus* [36], but this is not the case for many other bacterial infections. For example, the intracellular gram-positive bacterium *Listeria monocytogenes* induces a potent type I IFN response [37],[38], but *Ifnar*-deficient mice are actually more resistant to *L. monocytogenes* infection than are wildtype mice [39],[40],[41]. Many bacterial pathogens, including *Francisella tularensis*, *Mycobacterium tuberculosis*, *Brucella abortus*, and group B *Streptococcus*, induce type I IFN production by macrophages via a cytosolic TLR-independent pathway [42],[43],[44],[45], but the bacterial ligands and host sensors required for the interferon response of macrophages to these bacteria remain unknown.

It was demonstrated that induction of type I IFN by *L. pneumophila* in macrophages did not require bacterial replication or signaling through the TLR-adaptors MyD88 or Trif, but did require the bacterial Dot/Icm type IV secretion system [15]. Because the IFN response could be recapitulated with transfected DNA [15],[26] and because Dot/Icm system has been shown to conjugate DNA plasmids to recipient bacteria [46], it was proposed that perhaps *L. pneumophila* induced type I IFN via a cytosolic DNA-sensing pathway [15]. Another report used RNA interference to implicate the signaling adaptor IPS-1 (MAVS) in the IFN response to *L. pneumophila* in human A549 epithelial-like cells [14]. However, the significance of this latter finding is unclear since RNAi-mediated knockdown of RIG-I and MDA5, the two sensor proteins directly upstream of IPS-1, did not have an effect on induction of type I IFN by *L. pneumophila* [14]. Moreover, the A549 response to *L. pneumophila* may be distinct from the macrophage or *in vivo* response.

Recently, one report proposed that *L. pneumophila* DNA was recognized in the cytosol by RNA polymerase III [29], resulting in the production of an RNA intermediate that triggered IFN production via the IPS-1 pathway. Apparently consistent with this proposal, *Ips-1*-deficient mouse macrophages did not produce type I IFN in response to *L. pneumophila* [29]. Moreover, since Pol III acts preferentially on AT-rich substrates, it is plausible that Pol III would recognize the *L. pneumophila* genome, which has a high proportion (62%) of A:T basepairs. However, the response to *L. pneumophila* DNA was not investigated [29]. In addition, the same report, as well as others [28],[34], observed that the type I IFN response to AT-rich (or any other) DNA is not *Ips-1*-dependent in mouse cells. Thus, if *L. pneumophila* DNA was reaching the cytosol, the simplest prediction would be that the resulting type I IFN response would be independent of *Ips-1*, instead of *Ips-1*-dependent, as was shown [29]. Thus, the mechanism of IFN induction by *L. pneumophila* remains unclear.

In the present study, we sought to define bacterial and host factors controlling the macrophage type I IFN response to *L. pneumophila*. In agreement with previous studies [14],[29], we find that *Ips-1* is required for optimal induction of type I IFN in response to *L. pneumophila* infection *in vitro*. We extend this observation by demonstrating that *Ips-1* also contributes to the type I IFN response in an *in vivo* model of Legionnaires' Disease. Furthermore, we provide the first evidence that two RNA sensors upstream of *Ips-1, Rig-i* and *Mda5*, are involved in the macrophage interferon response to *L. pneumophila*. Importantly, however, we did not observe a role for the Pol III pathway in the type I IFN response to *L. pneumophila*. Instead, we found that *L. pneumophila* genomic DNA stimulates an *Ips-1*/*Mda5*/*Rig-i*-independent IFN response in macrophages, which contrasts with the *Ips-1*-dependent response to *L. pneumophila* infection. On the other hand, we found that *L. pneumophila* RNA stimulated a *Rig-i*-dependent IFN response. Thus, our data are consistent with a model in which *L. pneumophila* RNA, or host RNA, rather than *L. pneumophila* DNA, is the primary ligand that stimulates the host IFN response. We also investigated whether bacterial factors that modulate the host type I IFN response. Although numerous viral proteins that interfere with IFN signaling have been described [19], similar bacterial proteins have not been documented. It is therefore interesting that we were able to identify a secreted bacterial effector, SdhA, as an inhibitor of the *Ips-1*-dependent IFN response to *L. pneumophila*. Taken together, our findings provide surprising evidence that cytosolic RNA-sensing pathways are not specific for viral infections but can also respond to bacterial infections, and moreover, our data provide a specific example of a bacterial factor that suppresses the host IFN response.

## Results

### The cytosolic RNA-sensing pathway is involved in the macrophage response to *L. pneumophila*

**Figure 1:**
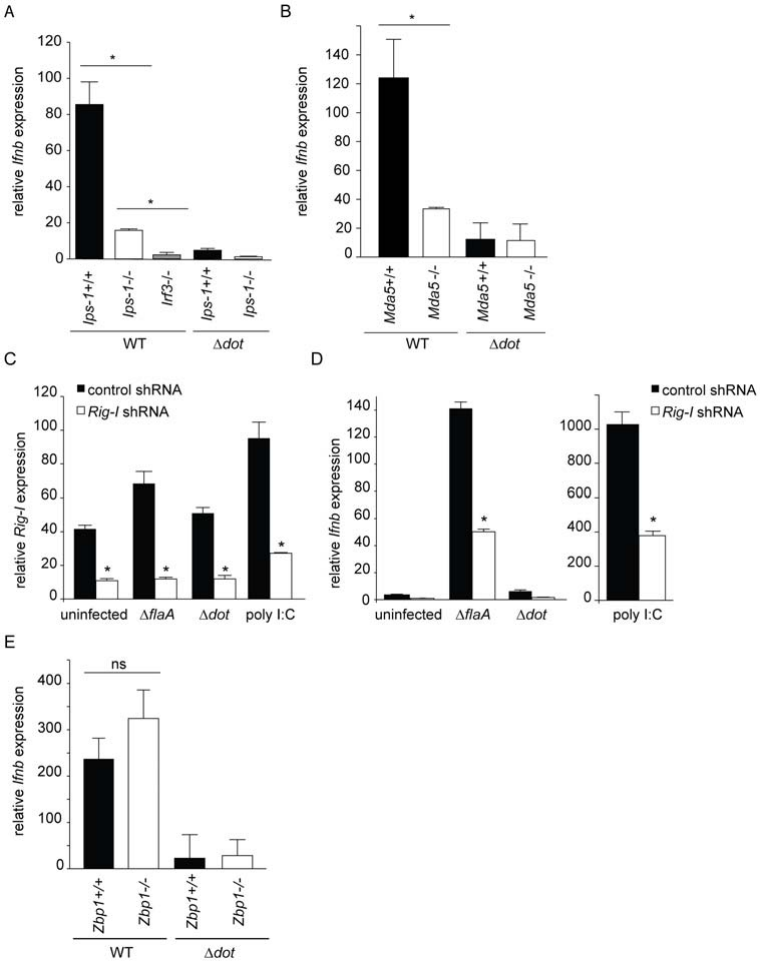
The cytosolic RNA-sensing pathway is involved in the host type I interferon response to *L. pneumophila*. (A) Induction of interferon beta (*Ifnb*) by *L. pneumophila* is largely dependent on *Ips-1*. Bone marrow derived *Ips-1^+/+^*, *Ips-1* ^−/−^, and *Irf3* ^−/−^ macrophages were infected with wild type and Δ*dot L. pneumophila* at a multiplicity of infection (MOI) of 1. *Ifnb* induction was analyzed by quantitative RT-PCR 4 hours post infection. *Ifnb* message was normalized to ribosomal protein *rps17* levels. Differences in *Ifnb* transcript induction were statistically significant in *Ips-1^+/+^*versus *Ips-1* ^−/−^ macrophages (*, p<0.05) and *Ips-1* ^−/−^ versus *Irf3* ^−/−^ (*, p<0.05, Student's t-test) when infected with wild type *L. pneumophila*. (B) Induction of *Ifnb* by *L. pneumophila* is partially dependent on *Mda5*. Bone marrow derived *Mda5* ^+/+^ and *Mda5* ^−/−^ macrophages were infected with wild type and Δ*dot L. pneumophila* at a multiplicity of infection (MOI) of 1. *Ifnb* induction was assessed by quantitative RT-PCR as in (A). Differences in *Ifnb* induction were statistically significant (*, p<0.05, Student's t-test) between *Mda5* ^+/+^ and *Mda5* ^−/−^ infected with wild type *L. pneumophila*. (C) Retroviral transduction of a *Rig-i* shRNA, but not the control shRNA, knocks down expression of *Rig-i* in *MyD88* ^−/−^ *Trif* ^−/−^ immortalized macrophages. Stable transduction of *MyD88* ^−/−^ *Trif* ^−/−^ immortalized macrophages was performed with a retroviral vector containing a control and *Rig-i* shRNA. Level of *Rig-i* knockdown was determined by quantitative RT-PCR under uninfected, infected, and poly I:C stimulation conditions. Differences in *Rig-i* transcript levels were statistically significant (*, p<0.05, Students t-test) under resting, infected, and ligand-stimulated conditions. (D) *Rig-i* is involved in the host type I interferon response to infection with *L. pneumophila*. *Rig-i* knockdown leads to reduced *Ifnb* expression in response to infection with Δ*flaA L. pneumophila*, as well as stimulation with poly I:C. Quantitative RT-PCR was carried out 4 hours post infection. Control knockdown macrophages induced a statistically significant (*, p<0.05) higher level of *Ifnb* transcript in response to Δ*flaA L. pneumophila* and poly I:C. No significant difference was found in uninfected or Δ*dot L. pneumophila* infected macrophages. (E) Induction of *Ifnb* by *L. pneumophila* is independent of *Zbp-1* (*Dai*). Bone marrow derived *Zbp-1^+/+^* and *Zbp-1* ^−/−^ macrophages were infected with *L. pneumophila* strains and analyzed for *Ifnb* induction as in (A) and (B). Differences in *Ifnb* transcript levels between *Zbp-1^+/+^* and *Zbp-1* ^−/−^ macrophages infected with *L. pneumophila* were not statistically significant (ns, p>0.1, Student's t-test).

We hypothesized that a cytosolic innate immune sensing pathway controls the type I IFN response to *L. pneumophila*. To test this hypothesis, we determined whether macrophages deficient in known cytosolic RNA and DNA sensing pathway components can induce type I IFNs in response to *L. pneumophila*. Macrophages were infected with *L. pneumophila* at a multiplicity of infection (MOI) of 1 and induction of interferon beta (*Ifnb*) message was analyzed by quantitative RT-PCR after 4 hours (Figure 1A–D). As previously reported [29], *Ips-1* ^−/−^ macrophages showed a significantly reduced induction of *Ifnb* in response to infection with wild type *L. pneumophila* compared to *Ips-1^+/+^* macrophages (p<0.05; Figure 1A). Induction of *Ifnb* was not completely eliminated in *Ips-1* ^−/−^ macrophages, however, as *Irf3* ^−/−^ macrophages exhibited an even lower induction of *Ifnb* compared to *Ips-1* ^−/−^ (p<0.05; Figure 1A). Consistent with previous reports [15], we found that the Dot/Icm type IV secretion system was required to elicit the macrophage type I interferon response since Δ*dot L. pneumophila* did not induce a robust type I interferon response (Figure 1A). These results suggest that *L. pneumophila* induces type I IFN *via* a cytosolic RNA immunosurveillance pathway that involves the adaptor Ips-1.

We hypothesized that a cytosolic RNA sensor that functions upstream of *Ips-1* could be involved in the type I interferon host response to *L. pneumophila*. However, knockdown experiments in A549 cells previously failed to reveal a role for the known sensors (MDA5 and RIG-I) upstream of IPS-1 [14]. Therefore, we tested *Mda5* ^−/−^ knockout macrophages (Figure 1B) and found reduced induction of *Ifnb* message as compared to control *Mda5^+/+^* macrophages. Importantly, however, *Dot*-dependent induction of type I IFN was not completely abolished in *Mda5* ^−/−^ macrophages, implying that other redundant pathways are also involved.

*Rig-i* knockout mice die as embryos, so we were unable to obtain *Rig-i* ^−/−^ knockout macrophages. To circumvent this problem, we stably transduced immortalized macrophages with a retrovirus expressing an shRNA to knock down *Rig-i* expression. Quantitative RT-PCR demonstrated that the knockdown was effective, even in infected macrophages (Figure 1C), and that *Rig-i* knockdown had a significant effect on the induction of type I interferon by *L. pneumophila* (Figure 1D). In the experiments in Figures 1C and 1D we used the Δ*flaA* strain of *L. pneumophila*, but similar results were obtained with wildtype, and it was previously shown that flagellin is not required for the IFN response to *L. pneumophila* [14],[16]. It is unusual, but not unprecedented, that a pathogen would stimulate both the RIG-I and MDA5 RNA-sensing pathways [47].

At present, only one candidate cytosolic DNA sensor involved in the IFN response has been described [30],[31]. To determine whether this sensor, called Dai (or Zpb1), is involved in the type I interferon response to *L. pneumophila*, we tested whether *Zbp1* ^−/−^ macrophages respond to *L. pneumophila*. We observed similar levels of *Ifnb* induction in *Zbp1^+/+^* and *Zbp1* ^−/−^ macrophages (Figure 1E). Taken together, these results imply that the RNA sensors Rig-i and Mda5, but not the DNA sensor Zbp1, are involved in sensing *L. pneumophila* infection.

We tested whether loss of signaling through the RNA sensing components *Ips-1* or *Mda5* could mimic the previously observed permissiveness of *Ifnar* ^−/−^ macrophages [16]. However, neither *Ips-1* ^−/−^ nor *Mda5* ^−/−^ macrophages were permissive to *L. pneumophila*, suggesting that the low levels of IFNβ produced in the absence of *Ips-1* or *Mda5* are sufficient to restrict *L. pneumophila* growth (Figure S1).

### The type IV secreted effector SdhA suppresses induction of interferon by *L. pneumophila*

To identify bacterial components that modulate the type I interferon response to *L. pneumophila*, we conducted a transposon mutagenesis screen. The LP02 strain of *L. pneumophila* was mutagenized with a *mariner* transposon as described previously [12]. Individual transposon mutants were used to infect *MyD88* ^−/−^ *Trif* ^−/−^ bone marrow-derived macrophages at an MOI of 1, and after approximately 16 hours, supernatants were collected and overlayed on type I IFN reporter cells [48]. Induction of type I IFN was compared to wild type (LP02) and Δ*dot L. pneumophila* controls. We tested approximately 2000 independent mutants and isolated eight mutants that were confirmed to be defective in induction of type I IFN. All these mutants harbored insertions in genes required for the function of the *Dot/Icm* apparatus (e.g., *icmB, icmC, icmD, icmX, icmJ*), thereby validating the screen.

**Figure 2:**
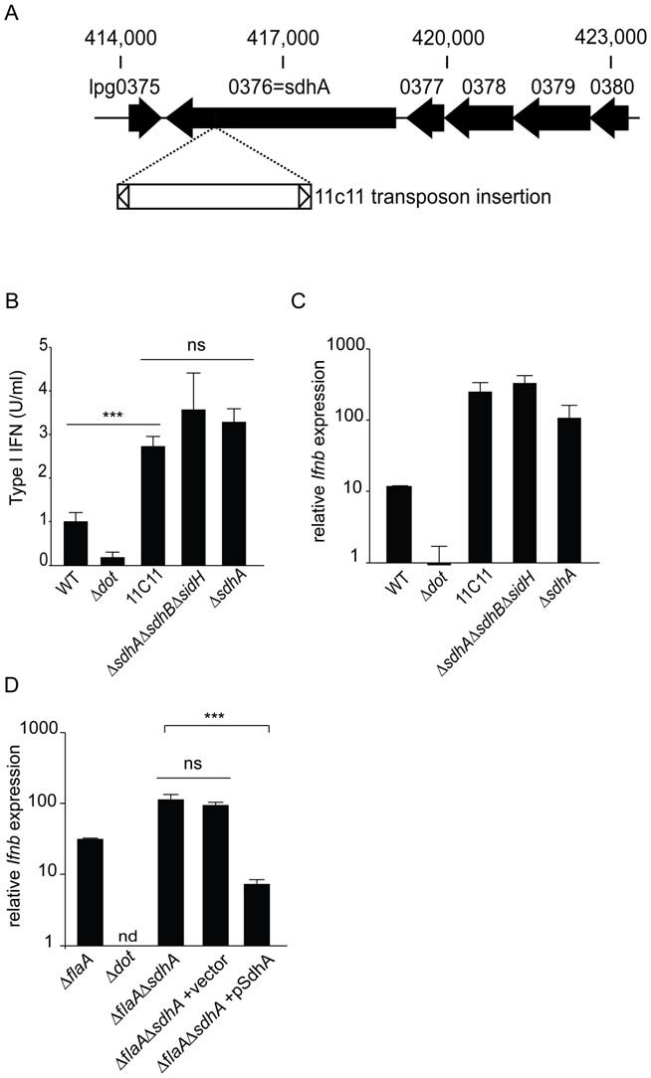
The type IV secreted effector SdhA suppresses induction of interferon by *L. pneumophila*. (A) The 11C11 mutant harbors a transposon insertion in *sdhA*. The transposon insertion site is in the 3′ end of the open reading frame of the *sdhA* locus at nucleotide position 3421. (B) A clean deletion mutant of *sdhA* recapitulates the 11C11 transposon mutant and hyperinduces type I interferon. Bone marrow derived *Myd88* ^−/−^ *Trif* ^−/−^ macrophages were infected with stationary phase *L. pneumophila* strains at a MOI of 1. Cell supernatants were harvested 8 hours post infection and assayed for type I interferon induction by an L929-ISRE luciferase bioassay. Type I interferon levels were determined by generating a standard curve with recombinant IFNβ An unmarked clean deletion of *sdhA* was compared to wild type, Δ*dot*, the transposon mutant 11C11, and a triple deletion of *sdhA* and the two *L. pneumophila* paralogs, *sidH* and *sdhB*. Differences in IFNβ induction were statistically significant between WT *L. pneumophila* and the transposon mutant 11C11 (***, p<0.0005, Student's t-test). Differences between 11C11, Δ*sdhA* and Δ*sdhA*Δ*sdhB*Δ*sidH* were not statistically significant (ns, p>0.05, Student's t-test). (C) A clean deletion mutant of *sdhA* recapitulates the 11C11 transposon mutant and hyperinduces transcriptional activation of *Ifnb*. Bone marrow derived *Myd88* ^−/−^ *Trif* ^−/−^ macrophages were infected with wild type, Δ*dot*, Δ*sdhA*, 11C11, Δ*sdhA*Δ*sdhB*Δ*sidH* stationary phase *L. pneumophila* and transcriptional induction of *Ifnb* was analyzed by quantitative RT-PCR. (D) Complementation of the *sdhA* mutant results in loss of the *Ifnb* hyperinduction phenotype. *MyD88* ^−/−^ *Trif* ^−/−^ BMDM were infected at an MOI of 1 with Δ*flaA*, Δ*dot*, Δ*flaA*Δ*sdhA* and Δ*flaA*Δ*sdhA L. pneumophila* carrying vector or a plasmid expressing full length SdhA. Expression of *Ifnb* message was assessed by quantitative RT-PCR 4 hours post infection.

Interestingly, a single transposon mutant, 11C11, was found that consistently hyperinduced the type I interferon response. The transposon insertion mapped to the 3′ end (nucleotide position 3421 of the open reading frame) of a gene, *sdhA*, that was previously shown [4] to encode a type IV secreted effector protein of 1429 amino acids (166kDa) (Figure 2A). SdhA has previously been shown to be essential for bacterial replication in macrophages [4], but a connection to type I IFNs was not previously noted.

To confirm that the hyperinduction of type I interferon was due to mutation of *sdhA*, the 11C11 transposon mutant was compared to an unmarked clean deletion of *sdhA* (Figure 2B). Both the 11C11 mutant and Δ*sdhA L. pneumophila* showed similar levels of hyperinduction of type I interferon. The *L. pneumophila* genome contains 2 paralogs of *sdhA*, called *sidH* and *sdhB*. A triple knockout strain, Δ*sdhA*Δ*sdhB*Δ*sidH,* was compared to single deletion of *sdhA* to determine if either paralog regulated the induction of type I IFNs. Similar levels of IFNβ were induced Δ*sdhA*Δ*sdhB*Δ*sidH* and Δ*sdhA* (Figure 2B). Similar results were obtained when induction of *Ifnb* was assessed by quantitative RT-PCR (Figure 2C). A role for *sdhA* in regulating the interferon response was further confirmed by complementing the Δ*sdhA* mutation with an *sdhA* expression plasmid [4]. As expected, the complemented strain induced significantly less type I IFN than the control Δ*sdhA* strain harboring an empty plasmid (Figure 2D). These results indicate that SdhA functions, directly or indirectly, to repress the induction of type I IFN by *L. pneumophila*.

### Hyperinduction of type I IFN by the *sdhA* mutant involves the cytosolic RNA-sensing pathway

**Figure 3:**
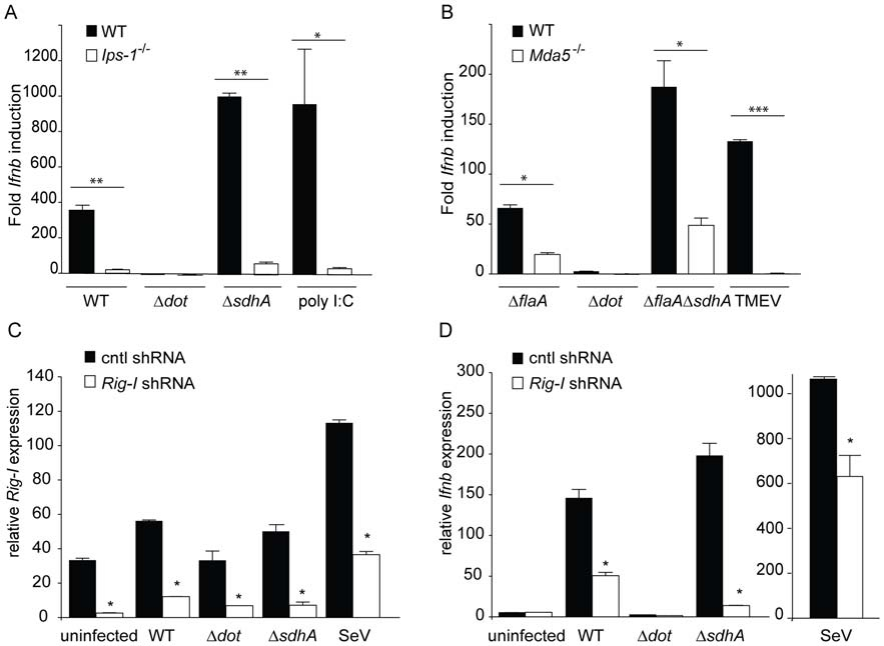
Hyperinduction of type I IFN by *sdhA* mutants involves cytosolic RNA sensing pathway components *Ips-1*, *Rig-i*, and *Mda5*. (A) Hyperinduction of *Ifnb* by Δ*sdhA L. pneumophila* is largely dependent on *Ips-1*. Bone marrow derived *Ips-1^+/+^* and *Ips-1* ^−/−^ macrophages were infected with wild type, Δ*dot*, and Δ*sdhA L. pneumophila* at an MOI of 1. *Ips-1^+/+^* and *Ips-1* ^−/−^ macrophages were transfected with 1.0 µg/ml poly I:C. 4 hours post infection and stimulation, macrophages were harvested and assessed for *Ifnb* induction as in Figure 1. *Ips-1^+/+^* infected with WT *L. pneumophila* induced statistically significant higher levels of *Ifnb* transcript than *Ips-1* ^−/−^ (**, p<0.005, Student's t-test). The same phenotype was seen in *Ips-1^+/+^* infected with Δ*sdhA L. pneumophila* (**, p<0.005) and transfected with poly I:C (*, p<0.05) when compared to *Ips-1* ^−/−^. (B) Hyperinduction of *Ifnb* by Δ*sdhA L. pneumophila* is partially dependent on *Mda5*. Bone marrow derived *Mda5^+/+^* and *Mda5* ^−/−^ macrophages were infected with Δ*flaA*, Δ*dot*, and Δ*flaA*Δ*sdhA L. pneumophila* at an MOI of 1. Theiler's virus (TMEV) was overlaid onto *Mda5^+/+^* and *Mda5* ^−/−^ macrophages. 4 hours post bacterial and viral infection, macrophages were harvested and assessed for *Ifnb* induction by qRT-PCR as in Figure 1. *Ifnb* message was induced statistically significantly in *Mda5^+/+^* macrophages infected with Δ*flaA L. pneumophila* versus *Mda5* ^−/−^ (*, p<0.05, Student's t-test). *Mda5^+/+^* also responded statistically significantly to Δ*flaA*Δ*sdhA L. pneumophila* over *Mda5* ^−/−^ (*, p<0.05, Student's t-test), while Theiler's virus elicited a robust *Ifnb* response from *Mda5^+/+^* not seen in *Mda5* ^−/−^ (***, p<0.005, Student's t-test). (C) Retroviral transduction of a *Rig-i* shRNA, but not the control shRNA, knocks down expression of *Rig-i. MyD88* ^−/−^ *Trif* ^−/−^ immortalized macrophages were stably transduced with retroviral vector containing a control and *Rig-i* shRNA. Level of *Rig-i* knockdown was determined by quantitative RT-PCR under uninfected and infected conditions. Differences in *Rig-i* transcript levels were statistically significant (*, p<0.05, Students t-test) under resting and infected conditions. (D) *Rig-i* is involved in the hyperinduction of type I interferon by Δ*sdhA L. pneumophila*. *Rig-i* knockdown leads to reduced *Ifnb* expression in response to infection with WT and Δ*sdhA L. pneumophila*, as well as Sendai virus. Quantitative RT-PCR was carried out 4 hours post infection. Control knockdown macrophages induced a statistically significant (*, p<0.05) higher level of *Ifnb* transcript in response to WT and Δ*sdhA L. pneumophila* and Sendai virus. No significant difference was found in uninfected or Δ*dot L. pneumophila* infected macrophages.

It was possible that Δ*sdhA* mutants hyperinduced type I IFN via a pathway distinct from the normal cytosolic RNA-sensing pathway that responds to wildtype *L. pneumophila*. Therefore, to determine whether hyperinduction of type I interferon by Δ*sdhA* occurs through the same pathway that responds to wild type *L. pneumophila*, we infected *Ips-1* ^−/−^ and *Mda5* ^−/−^ macrophages with Δ*sdhA L. pneumophila.* Induction of *Ifnb* message was determined by quantitative RT-PCR. The hyperinduction of *Ifnb* seen in *Ips-1^+/+^* macrophages was almost abolished in *Ips-1* ^−/−^ macrophages (p<0.001; Figure 3A). As a control, induction of *Ifnb* by poly I:C, a double-stranded synthetic RNA analog, was also *Ips-1*-dependent as expected. Similarly, the hyperinduction of *Ifnb* was also reduced in *Mda5* ^−/−^ macrophages (p<0.01; Figure 3B). However, the *Mda5* ^−/−^ macrophages still induced significant amounts of *Ifnb,* suggesting that the requirement for *Mda5* is not complete. We also tested the Δ*sdhA* mutant in *Rig-i* knockdown macrophages. Rig-i knockdown appeared to be effective (Figure 3C) and specifically diminished *Ifnb* expression (Figure 3D). Thus, the residual *Ifnb* induction in *Mda5* ^−/−^ may be due to *Rig-i,* or to another uncharacterized pathway. As a control, Theiler's virus (TMEV) induced *Ifnb* in a completely *Mda5*-dependent manner, as expected (Figure 3B).

### The effects of the Δ*sdhA* mutant are independent of caspase-1 activation

**Figure 4:**
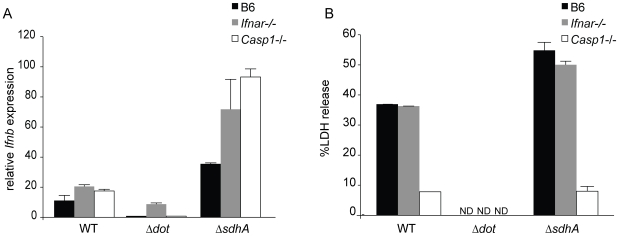
The effects of the *sdhA* mutation on type I interferon induction are independent of caspase-1 activation and the type I interferon receptor. (A) Type I interferon receptor signaling and caspase1-dependent pyroptotic cell death are not required for superinduction of *Ifnb* by the *sdhA* mutant. Bone marrow derived C57BL/6, *Ifnar* ^−/−^ and *Casp1* ^−/−^ macrophages were infected with wild type, Δ*dot*, and Δ*sdhA L. pneumophila* at an MOI of 1. *Ifnb* message was analyzed by qPCR from macrophage RNA harvested 4 hours post infection. (B) Caspase1-dependent pyroptotic cell death occurs independently of the type I interferon receptor. Bone marrow derived C57BL/6, *Ifnar* ^−/−^ and *Casp1* ^−/−^ macrophages were infected with wild type, Δ*dot*, and Δ*sdhA L. pneumophila* at an MOI of 1 and release of lactate dehydrogenase (LDH) in cell supernatants was measured 4 hours post infection. Specific cell lysis was calculated as a percentage of detergent lysed cells with spontaneous LDH release subtracted. No statistically significant difference was found between B6 and *Ifnar* ^−/−^ macrophages infected with Δ*sdhA L. pneumophila* (p>0.1, Student's t-test). ND, not detected.

It was previously shown that Δ*sdhA* mutants induce a rapid death of infected macrophages that is dependent upon activation of multiple cell death pathways [4]. Consequently, we hypothesized that the hyperinduction of type I IFN by the Δ*sdhA* mutant might be due to the release of molecules from dying cells, such as DNA, that could induce *Ifnb* expression. To rule out this explanation, we infected *Casp1* ^−/−^ macrophages, which are resistant to cell death at the early timepoints examined (e.g., 4h post infection), and asked whether type I interferon was still hyperinduced in response to Δ*sdhA L. pneumophila*. In fact, we found that *Casp1* ^−/−^macrophages infected with the Δ*sdhA* mutant hyperinduced *Ifnb* to levels above that observed in B6 macrophages (Figure 4A). We suspect that the increased *Ifnb* induction seen in *Casp1* ^−/−^ cells was an indirect consequence of the lower levels of cell death in these cells, and was not due to a specific suppression of type I interferon transcription by *Casp1* activation. In any case, our results indicated that the hyperinduction of type I IFN by the Δ*sdhA* mutant was not due to increased cell death induced by the mutant. As a control, we confirmed that *Casp1* ^−/−^ macrophages were resistant to cell death at the 4h timepoint tested (Figure 4B).

### SdhA acts independently of the type I IFN receptor

Induction of *Ifnb* is often regulated by a positive feedback loop in which initial production of IFNβ results in signaling through the type I IFN receptor (*Ifnar*) and synergistically stimulates the production of additional type I IFN. We therefore examined whether the hyperinduction of *Ifnb* by the Δ*sdhA* mutant might be due to positive feedback through the type I IFN receptor. To test this possibility we examined induction of *Ifnb* by the Δ*sdhA* mutant in *Ifnar* ^−/−^ macrophages. We found that hyperinduction of *Ifnb* by Δ*sdhA L. pneumophila* occurs even in the absence of signaling from the type I interferon receptor, since *Ifnar* ^−/−^ macrophages hyperinduce *Ifnb* in response to infection with Δ*sdhA L. pneumophila* (Figure 4A).

The mechanism by which the Δ*sdhA* mutant induces cell death remains unclear [4]. Studies with the intracellular bacterial pathogen *Francisella tularensis* have demonstrated the existence of a type I IFN-inducible caspase-1-dependent cell death pathway [43]. Therefore, we sought to establish if caspase-1-dependent cell death occurred in the absence of Ifnar signaling in response to wild type and Δ*sdhA L. pneumophila*. *Ifnar* ^−/−^ macrophages were infected at an MOI of 1 and assayed for release of the intracellular enzyme lactate dehydrogenase (LDH) 4 hours post infection. *Ifnar* ^−/−^ macrophages exhibited similar LDH release as B6 macrophages, whether infected with WT or Δ*sdhA L. pneumophila*, and this LDH release was dependent upon caspase-1 activation (Figure 4B). These data demonstrate that caspase1-dependent pyroptotic death occurs independently of the type I interferon receptor during infection with wild type and Δ*sdhA L. pneumophila*.

Since growth of the Δ*sdhA* mutant is severely attenuated in macrophages [4], we hypothesized that hyperinduction of type I interferon might contribute to the restriction of replication of the Δ*sdhA* mutant. To test this hypothesis, we infected *lfnar* ^−/−^ macrophages with luminescent strains of *L. pneumophila* at an MOI of 0.01 and monitored bacterial replication over a 72 hour time period. As previously reported [16], *lfnar* ^−/−^ macrophages were more permissive to WT and Δ*flaA L. pneumophila* as compared to C57BL/6 macrophages (Figure S2A, C). However, the Δ*sdhA* or Δ*flaA*Δ*sdhA L. pneumophila* strains were still significantly restricted in *Ifnar* ^−/−^ macrophages (Figure S2B, D). Thus, SdhA is required for bacterial replication in macrophages primarily via a mechanism independent of its role in suppressing type I IFN. As expected, Δ*dot L. pneumophila* did not replicate in WT or *Ifnar* ^−/−^ macrophages (Figure S2E).

Since SdhA is a secreted effector, we hypothesized that SdhA may act in the host cell cytosol, rather than in the bacterium, to repress *Ifnb* induction. To test this hypothesis, we co-expressed SdhA with MDA5 or RIG-I, by transient transfection of HEK293T cells, and assessed interferon expression with an IFNβ-luciferase reporter. Expression of either MDA5 or RIG-I robustly induced the IFNβ-luc reporter upon stimulation with poly I:C (Figure S3). When SdhA was co-expressed with MDA5, a dose-dependent repression of the IFNβ-luc reporter was observed (Figure S3A). Co-expression of SdhA also resulted in a dose-dependent repression of RIG-I-dependent induction of the IFNβ-luc reporter (Figure S3B). However, SdhA co-expression did not affect TRIF-dependent induction of the IFNβ-luc reporter (Figure S3C), arguing against the possibility that SdhA expression has non-specific effects on IFNβ-luc induction. These results must be interpreted with caution since the 293T IFNβ-luc reporter system is highly artificial; moreover, we have not demonstrated a direct interaction of SdhA with signaling components in the RNA-sensing pathway. In fact, the reported effects of SdhA on mitochondria [4] suggest the effect may be somewhat indirect (see Discussion). Nevertheless, the 293T transfection results suggest that SdhA can act in the host cytosol to specifically repress induction of the RIG-I/MDA5 pathway.

### *L. pneumophila* genomic DNA does not appear to stimulate an *Ips-1*-dependent IFN response

**Figure 5:**
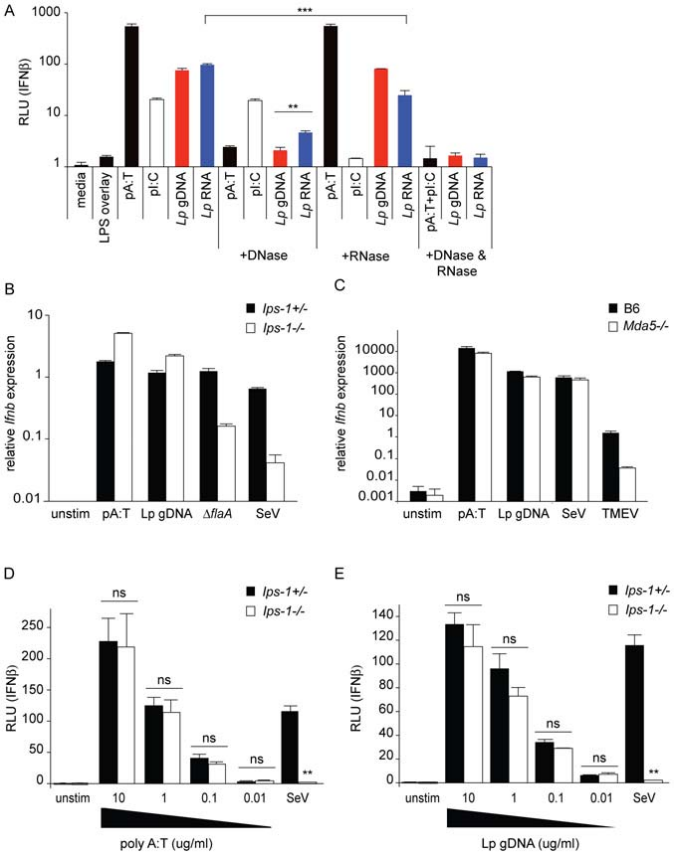
*L. pneumophila* DNA and RNA stimulate type I IFN production in macrophages. (A) Purified genomic DNA and RNA from *L. pneumophila* induces type I interferon independently of *MyD88* and *Trif*. Bone marrow derived *Myd88* ^−/−^ *Trif* ^−/−^ macrophages were stimulated by transfection of 3.3 µg/ml purified *L. pneumophila* DNA, *L. pneumophila* RNA, pA:T (DNA), and pI:C (RNA). Nucleic acids were treated with DNase and/or RNase A before transfection. Macrophage supernatants were harvested 8 hours post stimulation and analyzed for IFNβ levels by L929-ISRE luciferase bioassay. IFNβ production by DNase-treated *L. pneumophila* RNA was statistically significantly higher compared to DNase-treated *L. pneumophila* DNA (**, p<0.005). In addition, RNase A-treated *L. pneumophila* RNA produced statistically significant lower levels of IFNβ (***, p<0.0001, Student's t-test) than *L. pneumophila* RNA and RNase-treated *L. pneumophila* DNA. (B) Genomic *L. pneumophila* DNA does not induce type I interferon in a *Ips-1*-dependent manner. *Ips-1* ^−/−^ and heterozygous littermate bone marrow derived macrophages were stimulated by transfection of 1.0 µg/ml pA:T and purified genomic *L. pneumophila* DNA. Macrophages were infected with Δ*flaA L. pneumophila* at an MOI of 1. Sendai virus (SeV) was overlaid onto *Ips-1* ^−/−^ and heterozygous littermate macrophages. Transcriptional activation of *Ifnb* was determined by quantitative RT-PCR as described in Figure 1. (C) The viral RNA sensor *Mda5* is not required for induction of type I interferon by *L. pneumophila* DNA. WT (C57BL/6) and *Mda5* ^−/−^ bone marrow derived macrophages were stimulated by transfection of 1.0 µg/ml pA:T and purified genomic *L. pneumophila* DNA. Sendai virus (SeV) and Theiler's virus (TMEV) were overlaid onto WT and *Mda5* ^−/−^ macrophages. Quantitative RT-PCR was used to determine *Ifnb* gene expression. (D) Non-CpG containing DNA (pA:T) does not induce *Ips-1-*dependent *Ifnb* at all concentrations tested. *Ips-1* ^−/−^ and heterozygous littermate bone marrow derived macrophages were stimulated with a titration of pA:T by transfection of 10, 1.0, 0.1, 0.01 µg/ml pA:T. The difference between *Ips-1* ^+/−^ and *Ips-1* ^−/−^ macrophages transfected with pA:T was not statistically significant (ns, p>0.1, Student's t-test). Sendai virus (SeV) was overlaid onto *Ips-1* ^−/−^ and heterozygous littermate macrophages (**, p<0.005). Cell supernatants were collected 8 hours post stimulation/infection. Induction of type I interferon was determined by L929-ISRE luc bioassay. Units are relative light units (RLU). (E) Genomic *L. pneumophila* DNA induces type I interferon independently of *Ips-1* at all concentrations tested. *Ips-1* ^−/−^ and heterozygous littermate bone marrow derived macrophages were stimulated with a titration of purified genomic *L. pneumophila* DNA by transfection of 10, 1.0, 0.1, 0.01 µg/ml *L. pneumophila* DNA. No statistically significant difference was found between *Ips-1* ^+/−^ and *Ips-1* ^−/−^ macrophages transfected with genomic *L. pneumophila* DNA (ns, p>0.1, Student's t-test). Sendai virus (SeV) was overlaid onto *Ips-1* ^−/−^ and heterozygous littermate controls (**, p<0.005). Macrophage supernatants were collected 8 hours post stimulation/infection. Type I interferon levels were determined by L929-ISRE luc bioassay, units are relative light units (RLU).

Based on our observation that the host type I IFN response requires the *L. pneumophila* Dot/Icm type IV secretion system and was at least partly *Ips-1*, *Rig-i*, and *Mda5*-dependent, we hypothesized that *L. pneumophila* nucleic acids (RNA, DNA or both) might gain access to the macrophage cytosol *via* the type IV secretion system and induce a host type I interferon response. To test if *L. pneumophila* nucleic acids are sufficient to induce type I interferon, we transfected *MyD88* ^−/−^ *Trif* ^−/−^ macrophages with purified *L. pneumophila* genomic DNA or total RNA and determined the induction of type I interferons by bioassay. Poly(dA-dT):poly(dA-dT) (abbreviated as pA:T) was used as a non-CpG containing DNA control and poly I:C was used as an RNA control. Nucleic acid preparations were treated with DNase and/or RNase to eliminate contaminating nucleic acids. Both purified *L. pneumophila* DNA and the crude RNA preparation induced IFNβ (Figure 5A). *L. pneumophila* RNA treated with RNase also induced IFNβ, presumably due to (contaminating) DNA in the preparation (Figure 5A). However, *L. pneumophila* RNA treated with DNase induced type I interferon to a level above that induced by *L. pneumophila* RNA treated with both RNase and DNase, suggesting that *L. pneumophila* RNA alone can induce type I interferon production (Figure 5A). The induction of type I IFN by *L. pneumophila* RNA was modest, possibly because bacterial RNA is less stable than DNA. Nevertheless, these results suggest that both *L. pneumophila* RNA and DNA can induce a type I interferon host response.

Next, we determined if *L. pneumophila* nucleic acids could induce type I interferon in an *Ips-1*-dependent manner in macrophages. In certain cell types, though not mouse macrophages [34], AT-rich DNA has been shown to induce type I IFN via IPS-1 [26],[27],[28],[29]. It was important to assess whether *L. pneumophila* DNA, in particular, might signal in an *Ips-1*-dependent manner since the *L. pneumophila* type IV secretion system has previously been shown to translocate DNA [46]. *Ips-1* ^+/−^ and *Ips-1* ^−/−^ macrophages were transfected with pA:T and *L. pneumophila* DNA, as well as infected with Sendai virus, a virus previously determined to induce an *Ips-1*-dependent IFN response. Stimulation with pA:T or *L. pneumophila* DNA failed to induce *Ifnb* in an *Ips-1*-dependent manner, whereas Sendai virus induced significantly more *Ifnb* in *Ips-1* ^+/−^ versus *Ips-1* ^−/−^ macrophages (Figure 5B). Similar results were obtained in *Mda5* ^−/−^ macrophages: induction of type I IFN with pA:T or *L. pneumophila* genomic DNA showed no requirement for *Mda5*, whereas a control simulation, Theiler's Virus, showed *Mda5*-dependent induction of IFNβ, as expected (Figure 5C). It was possible that at high concentrations of DNA, an *Ips-1*-independent DNA-sensing pathway overwhelmed any putative *Ips-1*-dependent recognition of DNA. However, induction of *Ifnb* was independent of *Ips-1* even when titrated amounts of pA:T or *L. pneumophila* genomic DNA were transfected into macrophages (Figure 5D, 5E). Thus, these results suggest that while transfected *L. pneumophila* DNA robustly induces type I interferon, *L. pneumophila* genomic DNA does not appear to induce the *Ips-1*-dependent IFN response that is characteristic of *L. pneumophila* infection.

### *L. pneumophila* RNA stimulates type I interferon *via Rig-i*

**Figure 6:**
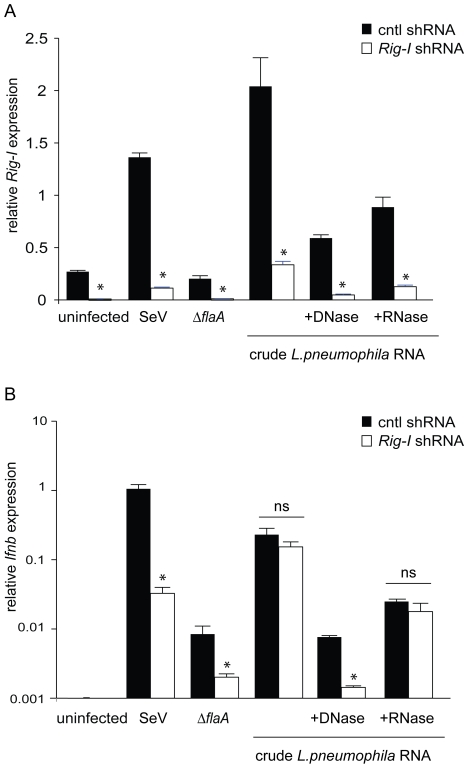
*L. pneumophila* RNA induces type I interferon *via Rig-i*. (A) The efficiency of *Rig-i* knockdown was determined by quantitative RT-PCR under uninfected, viral and bacterial infected, and transfected conditions. Differences in *Rig-i* transcript levels were statistically significant (*, p<0.05, Students t-test) under resting, infected, and transfected conditions. (B) *Rig-i* is involved in the host type I interferon response to *L. pneumophila* RNA. *Rig-i* knockdown leads to reduced *Ifnb* expression upon transfection with DNase-treated *L. pneumophila* RNA. Quantitative RT-PCR was carried out 4 hours post stimulation. Control knockdown macrophages induced a statistically significant (*, p<0.05) higher level of *Ifnb* transcript in Δ*flaA L. pneumophila* and Sendai virus infected macrophages. No significant difference was found in response to untreated and RNase-treated *L. pneumophila* nucleic acids.

To determine whether *L. pneumophila* RNA could be recognized by Rig-i, we transfected *L. pneumophila* RNA into macrophages in which *Rig-i* expression had been stably knocked down. Importantly, the *Rig-i* knockdown was performed in immortalized bone-marrow-derived macrophages that lack MyD88 and Trif, in order to avoid potential activation of known RNA-sensing TLRs. Knockdown of *Rig-i* was effective under our transfection conditions, as *Rig-i* message was significantly lower in macrophages transduced with a *Rig-i* shRNA compared to a control shRNA (p<0.05; Figure 6A). Crude *L. pneumophila* RNA (which also contains genomic DNA contaminants) induced *Ifnb* robustly in both control shRNA and *Rig-i* shRNA macrophages, even upon treatment with RNase A (Figure 6B). However, transfection of DNase-treated *L. pneumophil*a nucleic acids induced significantly less *Ifnb* in *Rig-i* knockdown macrophages as compared to control knockdown macrophages (p<0.05; Figure 6B.) This result suggests that *L. pneumophila* RNA can induce *Rig-i-*dependent type I interferon. It was not possible to perform a similar experiment in the *Ips-1* ^−/−^ macrophages because these macrophages were MyD88/Trif^+^ and exhibited background interferon, presumably due to TLR3 signaling.

### RNA polymerase III does not appear to be required for the IFN response to *L. pneumophila*

**Figure 7:**
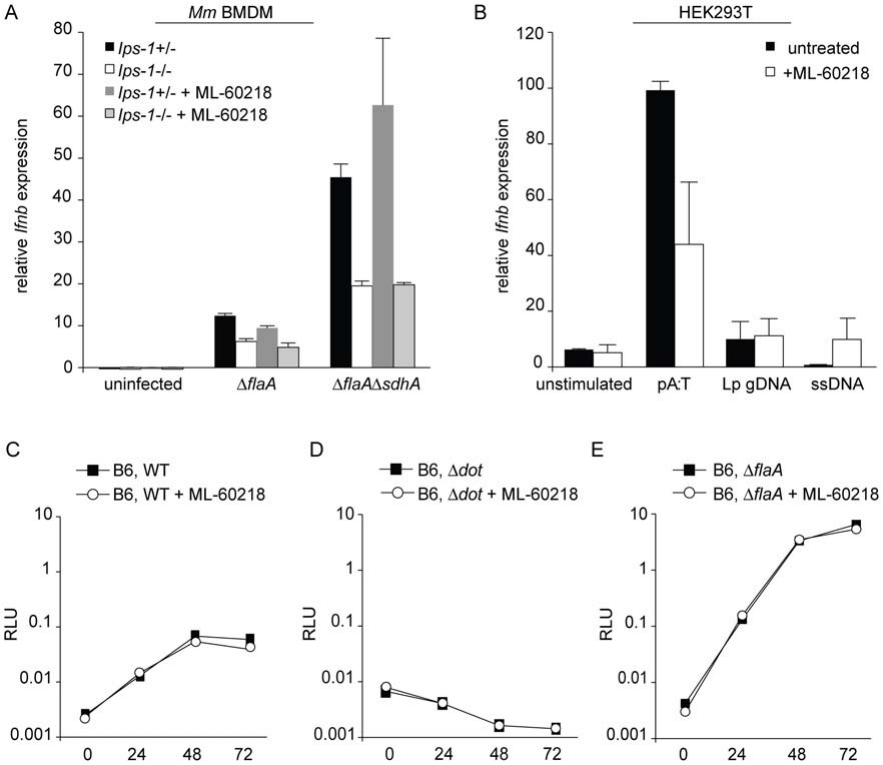
The Pol III pathway does not appear to recognize *L. pneumophila* DNA or affect *L. pneumophila* replication. (A) Inhibition of Pol III had no effect on *Ips-1*-dependent *Ifnb* induction by *L. pneumophila*. *Ips-1* ^+/−^ and *Ips-1* ^−/−^ macrophages were pretreated (controls were untreated) with 20 µM ML-60218 10 hours before infection with Δ*flaA* and Δ*flaA*Δ*sdhA L. pneumophila* at an MOI of 1. *Ifnb* induction was analyzed by quantitative RT-PCR 4 hours post infection. *Ifnb* message was normalized to ribosomal protein *rps17* levels. (B) *L. pneumophila* genomic DNA does not induce *IFNB* in HEK293T cells. HEK293T cells were pretreated, or left untreated, with 20 µM ML-60218 10 hours before transfection with 1.0 µg/ml pA:T, *L. pneumophila* genomic DNA, or salmon sperm DNA. *Ifnb* induction was analyzed by quantitative RT-PCR 4 hours post infection. *Ifnb* message was normalized to S9 levels. (C) WT (C57BL/6) macrophages were infected at an MOI of 0.01 in the presence or absence of 20 µM ML-60218 and growth of luminescent *L. pneumophila* strains was determined by RLU at 0, 24, 48, and 72 hours post infection. For inhibitor conditions, macrophages were pretreated with 20 µM ML-60218 10 hours before infection. Macrophages were infected with WT (LP02) *L. pneumophila* or with isogenic Δ*dot L. pneumophila* (D) Δ*flaA L. pneumophila* (E).

A recent report found that an inhibitor of RNA polymerase III, ML-60218 [49], blocked the type I IFN response to *L. pneumophila* [29]. It was proposed that *L. pneumophila* DNA is translocated into macrophages and transcribed by Pol III into a ligand that could be recognized by RIG-I [29]. In contrast, we did not see an effect of ML-60218 on induction of type I IFN by *L. pneumophila* in bone marrow-derived macrophages (Figure 7A). The lack of an effect does not appear to be due to redundant recognition by another DNA sensor in macrophages because the interferon induction was still largely *Ips-1*-dependent (Figure 7A). Because our results with the Pol III inhibitor were negative, we cannot rule out the possibility that the Pol III inhibitor fails to function in macrophages. However, we also tested 293T cells, which express only the Pol III pathway for cytosolic recognition of DNA [28],[29]. As expected, 293T cells responded to pA:T in an ML-60218-inhibitable manner, but did not respond well to *L. pneumophila* genomic DNA (Figure 7B), again suggesting that *L. pneumophila* genomic DNA is not an efficient substrate for the Pol III pathway. The Pol III inhibitor also appeared to have little effect on *L. pneumophila* replication in bone-marrow macrophages (Figure 7C–E). This latter result was expected, since we found that even *Ips-1* ^−/−^ macrophages exhibit normal restriction of *L. pneumophila* replication (Figure S1), despite significantly reduced IFN induction.

### *In vivo* role of *Ips-1* in the host type I interferon response to *L. pneumophila*

**Figure 8:**
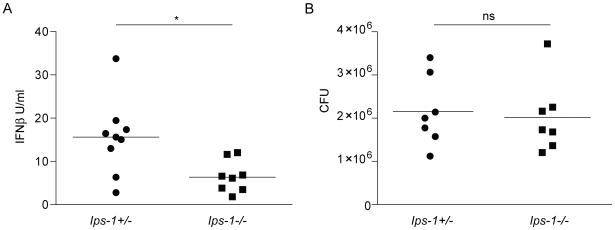
Role of *Ips-1* in the *in vivo* response to *L. pneumophila*. (A) The type I interferon response to *L. pneumophila* involves *Ips-1 in vivo*. *Ips-1* ^−/−^ and heterozygous littermate mice were infected intranasally with 2.5×10^6^ LP01Δ*flaA*. Bronchoalveolar lavage with PBS was performed 20 hours post infection. Type I interferon levels in the bronchoalveolar lavage fluid (BALF) were analyzed by bioassay and recombinant IFNβ was used to determine a standard curve. A two-tailed t-test determined the differences in IFNβ levels were statistically significant (*, p<0.01, Student's t-test) upon comparison of *Ips-1* ^+/−^ and *Ips-1* ^−/−^ mice. (B) *L. pneumophila* colony forming units are not significantly different in *Ips-1* ^+/−^ and *Ips-1* ^−/−^. Bronchoalveolar lavage fluid from infected *Ips-1* ^+/−^ and *Ips-1* ^−/−^ mice was centrifuged to isolate cells. Hypotonic lysis of cells was performed and CFU were plated on buffered yeast extract charcoal plates with antibiotic selection for *L. pneumophila*. A two-tailed t-test determined that CFU in *Ips-1* ^+/−^ and *Ips-1* ^−/−^ mice 20 hours post infection were not statistically significantly different (ns, p>0.5, Student's t-test).

In order to validate our findings *in vivo*, we infected *Ips-1* ^−/−^ and littermate *Ips-1* ^+/−^ mice with *L. pneumophila* (2.5×10^6^ LP01 Δ*flaA* per mouse, infected intranasally) and assayed type I interferon production in bronchoalveolar lavage fluid 20 hours post infection by bioassay. *Ips-1* ^+/−^ mice induced an IFN response that was statistically significantly greater than the response of *Ips-1* ^−/−^ mice (Student's t-test, p = 0.01; Figure 8A). The difference in IFN production was not explained by a difference in bacterial burden in the *Ips-1* ^+/−^ and *Ips-1* ^−/−^ mice, since both genotypes exhibited similar levels of bacterial colonization (p = 0.76, Student's t-test; Figure 8B). The lack of an effect of *Ips-1*-deficiency on bacterial replication *in vivo* was not surprising given that we also failed to observe an effect of *Ifnar*-deficiency on bacterial replication *in vivo* (data not shown). We suspect that type II IFN (IFNλ), which is not made by macrophages *in vitro,* or another *in vivo* pathway, may compensate for loss of type I IFN *in vivo*. Nevertheless, our results provided an important validation of our *in vitro* studies and affirm a role for *Ips-1* in the *in vivo* type I interferon response to *L. pneumophila*. Since *Ips-1*-deficient mice still mounted a measurable IFN response *in vivo*, it appears that additional *Ips-1*-independent pathways (e.g., TLR-dependent pathways, possibly involving other cell types [50]) also play a role *in vivo*.

## Discussion

Type I interferons (IFNs) have long been appreciated as critical players in antiviral immune defense, and recent work has identified several molecular immunosurveillance pathways that induce type I IFN expression in response to viruses [18],[19]. In contrast, the roles of type I IFNs in response to bacteria, and the pathways by which bacteria induce type I IFNs, are considerably less well understood. In this study, we sought to characterize the type I IFN response to the gram-negative bacterial pathogen *Legionella pneumophila*. Our study focused on the type I IFN response mounted by macrophages, since this is the cell type that is believed to be the primary replicative niche in the pathogenesis of Legionnaires' Disease.

In agreement with previous work [15], we found that *L. pneumophila* induces type I IFNs in macrophages *via* a TLR-independent pathway that requires expression of the bacterial type IV secretion system. These results suggested that a cytosolic immunosurveillance pathway controls the IFN response in macrophages. In this report we identify the cytosolic RNA-sensing pathway as a key responder to *L. pneumophila* infection (Figure 1) and, in agreement with previous results using human A549 cells [51], we did not observe a role for *Dai* (*Zbp1*), a gene implicated in the response to cytosolic DNA [30],[31]. A previous study using RNA interference in the human A549 epithelial-like cell line also found a role for IPS-1 in the type I IFN response to *L. pneumophila* [14]. However, knockdown of RIG-I or MDA5 did not appear to affect the IFN response [14], so the role of the IPS-1 pathway was unclear. In our study, we used mice harboring targeted gene deletions to establish a role for *Mda5* and *Ips-1* in the type I IFN response to *L. pneumophila* in macrophages, and uncovered a role for *Rig-i* using an shRNA knockdown strategy. We also found that the cytosolic RNA-surveillance pathway regulated the IFN response *in vivo* in a mouse model of Legionnaires' Disease.

After our manuscript was submitted, a report published by Chiu and colleagues also concluded that *Ips-1* is required for the macrophage type I IFN response to *L. pneumophila* [29]. However, the report of Chiu et al differs considerably from our current work by proposing that the type I IFN response to *L. pneumophila* occurs via a novel and unexpected pathway in which *L. pneumophila* DNA reaches the host cytosol and is transcribed by RNA polymerase III to generate an RNA intermediate that is sensed by RIG-I. Others have found that the Pol III pathway can be activated by viral and AT-rich DNA in certain cell types [28]. Our data, however, are not easily reconciled with a role for the Pol III pathway in recognition of *L. pneumophila*. First, and perhaps most important, is the observation that the response to DNA (in contrast to the response to *L. pneumophila* infection) has never been seen to be *Ips-1*-dependent in macrophages ([34]; Figure 5). This suggests that the response to *L. pneumophila* is not simply a response to DNA, regardless of the mechanisms by which potentially translocated DNA might be recognized.

We considered the possibility that *L. pneumophila* DNA exhibits unique properties that cause it to be a particularly efficient substrate for the Pol III pathway. Indeed, the *L. pneumophila* genome does contain stretches of highly AT-rich DNA, and it has been reported that only highly AT-rich DNA is an efficient substrate for the Pol III pathway [28],[29]. Therefore we tested whether *L. pneumophila* genomic DNA, unlike other DNA, could induce an *Ips-1*-dependent response in macrophages. Although *L. pneumophila* DNA induced a robust IFN response, the response was not *Ips-1*-dependent (Figure 5B, E). Indeed, even the optimal Pol III substrate poly(dA–dT):poly(dA–dT) (abbreviated as pA:T) does not appear to induce an *Ips-1*-dependent IFN response in macrophages (Figure 5B, D and [34]). The lack of *Ips-1*-dependence in the response to pA:T appears to be due to an unidentified *Ips-1*-independent DNA-sensing pathway that recognizes pA:T and dominates over the Pol III pathway in bone marrow macrophages [28]. Thus, if translocated DNA is the relevant bacterial ligand that stimulates the *Ips-1*-dependent host type I IFN response, an explanation is required for how the dominant and unidentified DNA-sensing pathway is not activated. While *L. pneumophila* could selectively inhibit or evade the dominant DNA-sensing pathway, there is at present no evidence to support this mechanism. Moreover, in our hands, the Pol III inhibitor used by Chiu et al (ML-60218) failed to affect IFN induction or bacterial replication in macrophages (Figure 7), in contrast to what would be predicted if the Pol III pathway was selectively activated in response to *L. pneumophila* infection. Therefore, our data lead us to consider alternative models.

Although Chiu et al primarily used the RAW macrophage-like cell line in their experiments with *L. pneumophila*, we do not believe that cell-type-specific effects can account for the discrepancy in results. Although it is possible that RAW cells express only the Pol III pathway, this would not change the fact that the proposed model of Chiu et al invokes DNA as the primary IFN-inducing ligand produced by *L. pneumophila*. The simplest prediction of such a model would be that the response of bone marrow macrophages to *L. pneumophila* would be *Ips-1*-*independent*, as is the response of macrophages to all forms of DNA that have been tested. In contrast, as documented here (Figure 1) and by Chiu et al [29], the response to *L. pneumophila* is *Ips-1-dependent*. Moreover, 293T cells, which express only the Pol III DNA-sensing pathway [28],[29], failed to respond significantly to *L. pneumophila* genomic DNA, despite a robust response to pA:T (Figure 7B). Therefore, our data suggest that recognition of *L. pneumophila* genomic DNA by Pol III is not responsible for the *Ips-1*-dependent IFN response to *L. pneumophila*.

We considered two other models to explain how *L. pneumophila* induces a type I interferon response. The first is that *L. pneumophila* translocates RNA into host cells. In support of this model, we demonstrate that *L. pneumophila* RNA, unlike any form of DNA tested, induced a *Rig-i-*dependent type I IFN response in macrophages (Figures 5A, 6). However, we did not demonstrate that *L. pneumophila* RNA species are translocated into host cells, and this will be important to examine in future studies. Interestingly, it was recently reported that purified *Helicobacter pylori* RNA stimulates RIG-I in transfected 293T cells [52]. A second model to explain type I IFN induction by *L. pneumophila* is that infection induces a host response that indirectly results in signaling via the MDA5/RIG-I/IPS-1 pathways. *L. pneumophila* secretes a large number of effectors into the host cytosol and these effectors disrupt or alter a large number of host cell processes [53]. Such disruption may either lead to the generation of host-derived RNA ligands for the RIG-I and MDA5 sensors, or may result in signaling through these sensors in the absence of specific ligands. It was previously proposed that a host nuclease, RNaseL, can generate self-RNA ligands for the RIG-I and MDA5 pathways in response to viral infection [54]. Although we could not observe a role for RNaseL in the response to *L. pneumophila* (K.M. Monroe, unpublished data), it is conceivable that a different host enzyme can fulfill a similar function.

Our finding that a secreted bacterial effector, SdhA, previously shown to suppress host cell death, also suppresses the IFN response to *L. pneumophila*, is consistent with a model in which a host cell stress response leads to direct or indirect activation of the cytosolic RNA-sensing pathway. However, the mechanism by which SdhA acts on host cells remains mysterious. Laguna and colleagues provided evidence that SdhA is critical for prevention of mitochondrial disruption that occurs when host cells are infected with the Δ*sdhA* mutant [4]. Given that Ips-1 localizes to mitochondria and requires mitochondrial localization for its function [21], it is tempting to speculate that SdhA acts on mitochondria in a way that both prevents their disruption and interferes with the function of Ips-1. To provide evidence that SdhA acts specifically on the RIG-I/MDA5 pathway, we used transient transfections of 293T cells. SdhA repressed induction of *Ifnb* when co-expressed with Mda5 or Rig-I but not Trif (Figure S3). Given these results and the evidence that SdhA is translocated into host cells [4], we favor the idea that SdhA acts within host cells. Mutation of *sdhA* was reported not to affect translocation of other effectors into host cells [4]; thus, we tend not to support the alternative possibility that SdhA blocks translocation of the putative IFN-stimulatory ligand through the type IV secretion system. SdhA is a large protein of 1429 amino acids, but does not contain domains of known function, except for a putative coiled coil (a.a. 1037–1068). In future studies it will be important to address whether subdomains of SdhA can be identified that are required for suppression of the IFN response. It will also be important to determine whether these subdomains are distinguishable from any putative subdomains required for suppression of host cell death. In fact, our data have suggested that suppression of cell death and the IFN response may be separable functions of SdhA. We found that cell death was not required for hyperinduction of IFN by the Δ*sdhA* mutant, and conversely, we also found that hyperinduction of type I IFN does not lead to increased cell death (Figure 4).

Our studies demonstrate a partial role for both *Mda5* and *Rig-i* RNA sensors in response to *L. pneumophila*. Although these sensors are typically thought to respond to distinct classes of viruses, there are indications that they can also function cooperatively in response to certain stimuli, e.g., West Nile Virus [47]. Our results suggest that *L. pneumophila* produces ligands that can stimulate both *Mda5* and *Rig-i* and that these two sensors cooperatively signal via *Ips-1*. Fitting with this model, we found that *Ips-1*-deficiency generally had a more severe impact on type I IFN induction than did *Mda5* or *Rig-i* deficiency.

Cytosolic RNA-sensing pathways are believed to respond exclusively to viral infection, and it is therefore surprising that *L. pneumophila* appears to trigger these pathways. Other bacterial species, such as *Listeria monocytogenes* and *Francisella tularensis,* have been shown to induce an *Ips-1*-independent cytosolic pathway leading to type I IFN induction [25],[43],[55]. The sensor(s) required for the IFN response to *Listeria* or *Francisella* have not yet been identified, but are widely assumed to be identical to the (also unknown) sensor(s) that respond to cytosolic DNA [15],[26].

*Ips-1* or *Mda5*-deficiency, as well as *Rig-i* knockdown, did not result in a complete elimination of the type I IFN response (Figure 1, Figure 3). Thus, a cytosolic DNA-sensing pathway may also be stimulated in response to *L. pneumophila* infection. A minor role for a cytosolic DNA-sensing pathway would be consistent with the observation that the *L. pneumophila* Dot/Icm type IV secretion system can translocate DNA into recipient cells [46]. However, as discussed above, our results with purified *L. pneumophila* DNA suggest that cytosolic sensing of *L. pneumophila* DNA does not account for the *Ips-1*-dependent induction of IFN that we observe (Figure 5). One last possibility that we cannot eliminate is that a non-DNA, non-RNA ligand is translocated into host cells and stimulates the *Ips-1* pathway. In fact, in separate work, we have found that a small bacterial cyclic dinucleotide, c-di-GMP, can trigger a type I IFN response in macrophages, but importantly, this response is entirely independent of the *Ips-1* pathway [34]. Nevertheless, there may be other small molecules that can be translocated by the Dot/Icm secretion system and signal in host cells via *Ips-1*.

Taken together, our results lead to new insights into the host immunosurveillance pathways that provide innate defense against bacterial pathogens. We demonstrate an unexpected role for a viral RNA-sensing pathway in the response to *L. pneumophila*, and identify a secreted bacterial effector, SdhA, that can suppress this response. Our results therefore open new possibilities for immunosurveillance of bacterial pathogens.

## Materials and Methods

### Ethics statement

Animal experiments were approved by the University of California, Berkeley, Institutional Animal Care and Use Committee.

### Mice, cell lines and plasmids

Bone marrow derived macrophages were derived from the following mouse strains: C57BL/6J (B6), *Ips-1* ^−/−^ [25], *Mda5* ^−/−^ [56], *Ifnar* ^−/−^ [57], *Zbp1* ^−/−^ [32], *MyD88/Trif* ^−/−^, and *Casp1* ^−/−^ [58]. C57BL/6J mice were purchased from the Jackson Laboratory. *Ips-1* ^−/−^ mice were from Z. Chen (University of Texas Southwestern Medical Center). *Ips-1* ^−/−^ were obtained on a mixed B6/129 background and *Ips-1* ^−/−^ and *Ips-1* ^+/−^ littermate controls were generated by breeding (*Ips-1* ^−/−^ x B6) F1 mice to *Ips-1* ^−/−^. *Mda5* ^−/−^ mice were from M. Colonna and S. Gilfillan (Washington University). L929-ISRE IFN reporter cells were from B. Beutler (The Scripps Research Institute). Viruses to immortalize *MyD88* ^−/−^ *Trif* ^−/−^ immortalized bone marrow derived macrophages were the generous gift of K. Fitzgerald, D. Golenbock (U. Mass, Worcester) and D. Kalvakolanu (U. Maryland). The complementation plasmid (pJB908-SdhA) was generously provided by R. Isberg (Tufts). Expression constructs pEF-BOS-RIG-I and pEF-BOS-MDA5 were generously provided by J. Jung (Harvard Medical School).

### Bacterial strains

LP02 is a streptomycin-resistant thymidine auxotroph derivative of *Legionella pneumophila* strain LP01. LP02Δ*sdhA* and LP02Δ*sdhA*Δ*sdhB*Δ*sidH* were a generous gift from R. Isberg (Tufts University). The Δ*flaA*Δ*sdhA* strain was generated by introducing an unmarked deletion of *flaA* in LP02Δ*sdhA* using the allelic exchange vector pSR47S-Δ*flaA* [12].

### Cell culture

L929-ISRE and HEK293T cells were cultured in DMEM supplemented with 10% FBS, 2 mM L-glutamine, 100 µM streptomycin, and 100 U/mL penicillin. Macrophages were derived from bone marrow cells cultured for eight days in RPMI supplemented with 10% FBS, 2 mM L-glutamine, 100 µM streptomycin, 100 U/mL penicillin, and 10% supernatant from 3T3-CSF cells, with feeding on the fifth day of growth. *MyD88* ^−/−^ *Trif* ^−/−^ immortalized macrophages were cultured in RPMI supplemented with 10% FBS, 2 mM L-glutamine, 100 µM streptomycin, and 100 U/mL penicillin.

### Reagents

Poly I:C was from GE Biosciences, pA:T (poly(dA-dT):poly(dA-dT)) was from Sigma, and Sendai Virus was from Charles River Laboratories. Wildtype Theiler's Virus GDVII was from M. Brahic and E. Freundt (Stanford University). Pol III inhibitor (ML-60218) was from Calbiochem.

### Isolation of nucleic acids from *L. pneumophila*

Total bacterial RNA was isolated using RNAprotect Bacterial Reagent (Qiagen) and RNeasy kit (Qiagen). Genomic DNA was isolated by guanidinium thiocyanate followed by phenol:chloroform extraction. Nucleic acids were treated with RQ1 RNase-Free DNase (Promega) and/or RNaseA (Sigma).

### Quantitative RT-PCR

Bone marrow derived macrophages were plated at a density of 2×10^6^ per well in 6 well plates and infected with an MOI of 1. Macrophage RNA was harvested 4 hours post infection and isolated with the RNeasy kit (Qiagen) according to the manufacturer's protocol. RNA was DNase treated with RQ1 RNase-Free DNase (Promega) and reverse transcribed with Superscript III (Invitrogen). Quantitative PCR assays were performed on the Step One Plus RT PCR System (Applied Biosystems) with Platinum Taq DNA polymerase (Invitrogen) and EvaGreen dye (Biotium). Gene expression values were normalized to *Rps17* (mouse) or S9 (human) levels for each sample. The following primer sequences were used: mouse *Ifnb*, F, 5′-ATAAGCAGCTCCAGCTCCAA-3′and R, 5′-CTGTCTGCTGGTGGAGTTCA-3′; mouse *Rps17*, F, 5′-CGCCATTATCCCCAGCAAG-3′ and R, 5′- TGTCGGGATCCACCTCAATG-3′; mouse *Rig-i,* F, 5′-ATTGTCGGCGTCCACAAAG-3′ and R, 5′-GTGCATCGTTGTATTTCCGCA-3′, human *Ifnb,* F, 5′-AAACTCATGAGCAGTCTGCA-3′ and R, 5′- AGGAGATCTTCAGTTTCGGAG G-3′; human S9, F, 5′-ATCCGCCAGCGCCATA-3′ and R, 5′-TCAATGTGCTTCTGGGAATCC-3′.

### Cell stimulation and transfection

Cell stimulants were transfected with Lipofectamine 2000 (LF2000, Invitrogen) according to the manufacturer's protocol. Nucleic acids were mixed with LF2000 in Optimem (Invitrogen) at a ratio of 1.0 µl LF2000/µg nucleic acid and incubated for 20 minutes at room temperature. The ligand-lipid complexes were added to cells at a final concentration of 3.3 µg/ml (96-well plates) and 1.0 µg/ml (6 well plates). For poly I:C, the stock solution (2.5 mg/ml) was heated at 55°C for 10 minutes and cooled to room temperature immediately before mixing with LF2000. Transfection experiments were incubated for 8 hours, unless otherwise stated. RIG-I, MDA5, TRIF and SdhA expression plasmids, along with an IFNβ-firefly luciferase reporter and TK-*Renilla* luciferase plasmids, were transfected with FuGENE 6 (Roche) according to the manufacturer's protocol. Nucleic acids were mixed with FuGENE 6 in Optimem at 0.5 µl/96 well and incubated for 15 minutes. Total transfected DNA was normalized to 200 ng per well using an empty pcDNA3 plasmid. Cells were stimulated 20 hours after transfection of expression plasmids.

### Type I IFN bioassay and luciferase reporter assay

Cell culture supernatants or bronchoalveolar lavage fluid (BALF) was overlayed on L929-ISRE IFN reporter cells in a 96-well plate format and incubated for 4 hours at 37°C and 5%CO_2_. L929-ISRE IFN reporter cells and HEK293T cells expressing an IFNβ-firefly luciferase reporter and TK-*Renilla* luciferase were lysed in Passive Lysis Buffer (Promega) for 5 minutes at room temperature and relative light units were measured upon injection of firefly luciferin substrate (Biosynth) or *Renilla* substrate with the LmaxII^384^ luminometer (Molecular Devices). For transient transfection reporter assays, luciferase values were normalized to an internal *Renilla* control.

### Cytotoxicity assays

Cytotoxicity of bacterial strains was determined by measuring lactate dehydrogenase release essentially as previously described [59]. Macrophages were plated at a density of 1×10^5^ in a 96-well plate and infected with stationary phase *L. pneumophila* at a multiplicity of infection (MOI) of 1. Plates were spun at 400×*g* for 10 minutes to allow equivalent infectivity of non-motile and motile strains [12]. Plates were re-spun 4 hours post infection and cell culture supernatants were assayed for LDH activity. Specific lysis was calculated as a percentage of detergent lysed cells.

### Growth curves

Bacterial growth was determined as previously described [16]. Bone marrow derived macrophages were plated at a density of 1×10^5^ per well in white 96-well plates (Nunc) and allowed to adhere overnight. Macrophages were infected with stationary-phase *L. pneumophila* at a multiplicity of infection (MOI) of 0.01. Growth of luminescent *L. pneumophila* strains was assessed by RLU with the LmaxII^384^ luminometer (Molecular Devices). Nonluminescent bacterial strains were analyzed for colony-forming units on buffered charcoal yeast extract plates.

### Transposon mutagenesis

Transposon mutagenesis of LP02 was previously described [12]. Briefly, the pSC123 mariner transposon was mated from *E.coli* SM10 λpir into the *L. pneumophila* strain LP02. Matings were plated on buffered yeast extract charcoal plates with streptomycin (100 µg/ml) and kanamycin (25 µg/ml). Single colonies were isolated and grown in overnight cultures and used to infect bone marrow derived *MyD88* ^−/−^ *Trif* ^−/−^ macrophages. After overnight incubation, levels of type I interferon in the supernatant was determined by bioassay. The site of transposon insertion was determined by Y-linker PCR [60].

### *In vivo* studies

Age and sex-matched *Ips-1* ^−/−^ and littermate *Ips-1* ^+/−^ mice were infected intranasally with 2.5×10^6^ LP01 Δ*flaA* in 20 µl PBS. Bronchoalveolar lavage was performed 20 hours post infection via the trachea using a catheter (BD Angiocath 18 g, 1.3×48 mm) and 800 µl PBS. Type I interferon induction was determined by bioassay. Type I interferon amounts were calculated using a 4-parameter standard curve determined by dilution of recombinant IFNβ (R&D Systems). CFUs were determined by hypotonic lysis of cells from the brochoalveolar lavage fluid (BALF). In parallel experiments, it was determined that CFU in the BALF was representative of total CFU in the lung.

### shRNA knockdown

Knockdown constructs were generated with the MSCV/LTRmiR30-PIG (LMP) vector from Open Biosystems. shRNA PCR products were cloned into the LMP vector using XhoI and EcoRI sites. *Rig-i* sequence: 5′-GCCCATTGAAACCAAGAAATT-3′, control shRNA sequence: 5′-TGACAGTGTCTTCGCTAATGAA-3′. *MyD88* ^−/−^ *Trif* ^−/−^ immortalized bone marrow derived macrophages were transduced with retrovirus as previously described [10]. GFP^+^ macrophages were sorted with the DAKO-Cytomation MoFlo High Speed Sorter.

## Supporting Information

Figure S1 *L. pneumophila* replication is restricted in *Ips-1* ^−/−^ and *Mda-5* ^−/−^ macrophages. *Ips-1* ^+/−^, *Ips-1* ^−/−^, C57BL/6 (B6) and *Mda5* ^−/−^ macrophages were infected at an MOI of 0.01 and growth of luminescent *L. pneumophila* strains was determined by RLU at 0, 24, 48, and 72 hours post infection. (A) *Ips-1* ^+/−^ and *Ips-1* ^−/−^ macrophages were infected WT (LP02) *L. pneumophila* (B) C57BL/6 (B6) and *Mda5* ^−/−^ macrophages were infected as in A (C) *Ips-1* ^+/−^ and *Ips-1* ^−/−^ macrophages were infected with Δ*dot L. pneumophila* (D) C57BL/6 (B6) and *Mda5* ^−/−^ macrophages were infected as in C (E) *Ips-1* ^+/−^ and *Ips-1* ^−/−^ infected with Δ*flaA L. pneumophila* (F) C57BL/6 (B6) and *Mda5* ^−/−^ macrophages were infected as in E.
(0.31 MB PDF)

Figure S2 Abrogation of type I interferon receptor signaling alone does not permit growth of Δ*sdhA* mutant. C57BL/6 (B6) and *Ifnar* ^−/−^ macrophages were infected at an MOI of 0.01 and growth of luminescent *L. pneumophila* strains was determined by RLU at 0, 24, 48, and 72 hours post infection. (A) C57BL/6 (B6) and *Ifnar* ^−/−^ macrophages were infected WT (LP02) *L. pneumophila* (B) macrophages were infected as in A but with Δ*sdhA L. pneumophila* (C) Δ*flaA L. pneumophila* (D) Δ*flaA*Δ*sdhA L. pneumophila* (E) Δ*dot L. pneumophila*.
(0.29 MB PDF)

Figure S3 SdhA represses MDA5 and RIG-I induction of interferon. Overexpression of SdhA in HEK293T cells results in repression of interferon induction mediated by MDA5 or RIG-I but not TRIF. (A) HEK293T cells were transfected with plasmids encoding the IFNβ-firefly luciferase reporter, TK-*Renilla* luciferase reporter (for normalization), full length MDA5 and/or increasing amounts of full length SdhA. At 20 hours post transfection, cells were transfected with poly I:C and then firefly luciferase and *Renilla* luciferase levels were determined 8 hours later. (B) Transfection and stimulation were performed as in A, except with a RIG-I expression plasmid and/or increasing amounts of full length SdhA expression plasmid. (C) Transfection and stimulation were performed as in A, except with a Trif expression plasmid and/or SdhA.
(0.27 MB PDF)
