# Supplementary figures and images for: Identification of Host Cytosolic Sensors and Bacterial Factors Regulating the Type I Interferon Response to Legionella pneumophila
Source: PLoS Pathog. 2009 Nov 20;5(11):e1000665. doi: 10.1371/journal.ppat.1000665 (PMC2773930; doi:10.1371/journal.ppat.1000665)

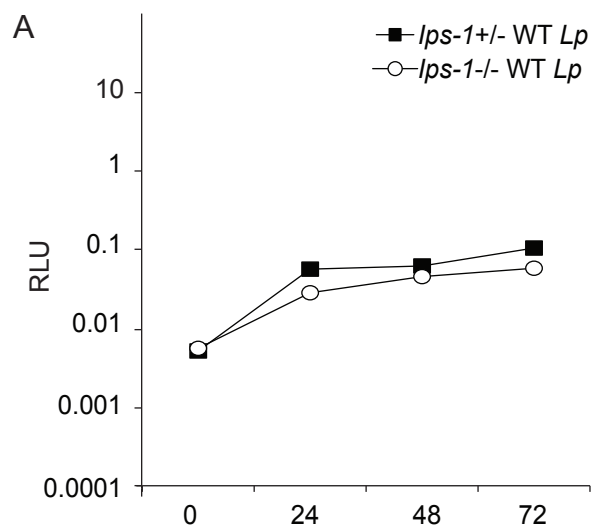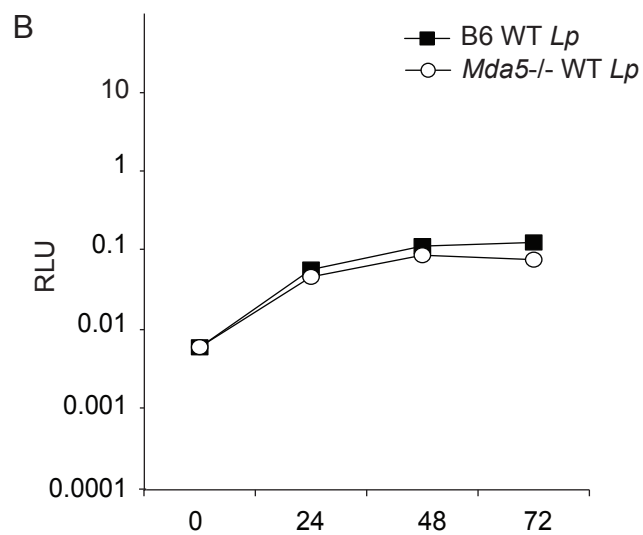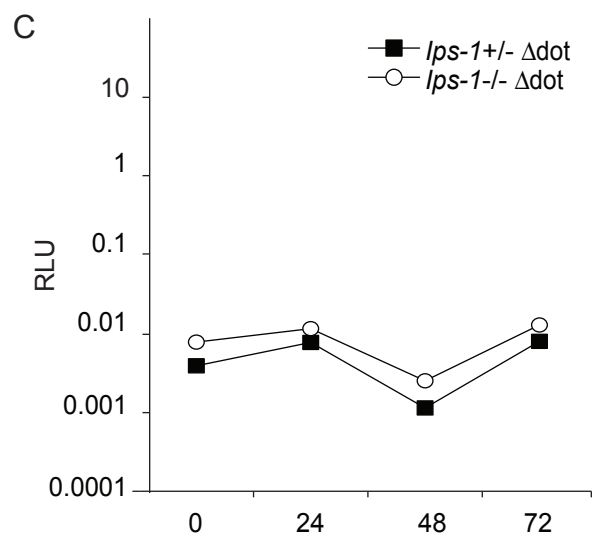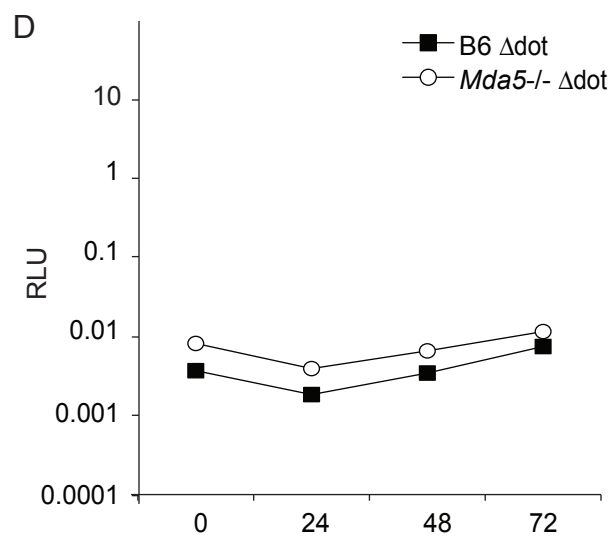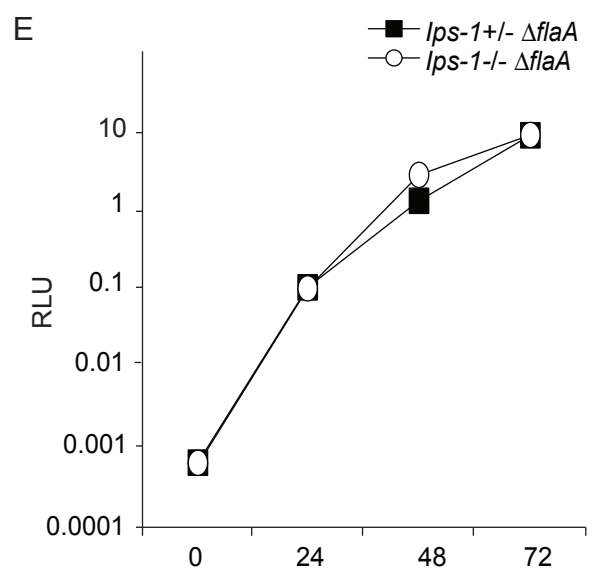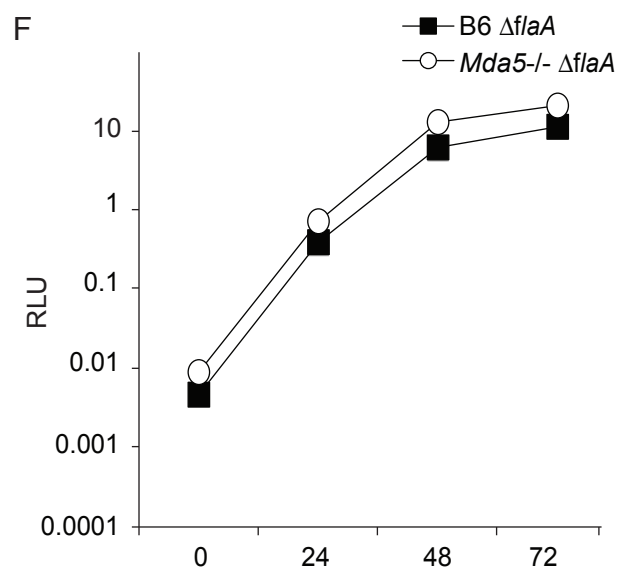

Supplement: Figure S1 — L. pneumophila replication is restricted in Ips-1 −/− and Mda-5 −/− macrophages. Ips-1 +/−, Ips-1 −/−, C57BL/6 (B6) and Mda5 −/− macrophages were infected at an MOI of 0.01 and growth of luminescent L. pneumophila strains was determined by RLU at 0, 24, 48, and 72 hours post infection. (A) Ips-1 +/− and Ips-1 −/− macrophages were infected WT (LP02) L. pneumophila (B) C57BL/6 (B6) and Mda5 −/− macrophages were infected as in A (C) Ips-1 +/− and Ips-1 −/− macrophages were infected with Δdot L. pneumophila (D) C57BL/6 (B6) and Mda5 −/− macrophages were infected as in C (E) Ips-1 +/− and Ips-1 −/− infected with ΔflaA L. pneumophila (F) C57BL/6 (B6) and Mda5 −/− macrophages were infected as in E. (0.31 MB PDF) [file ppat.1000665.s001.pdf]

A

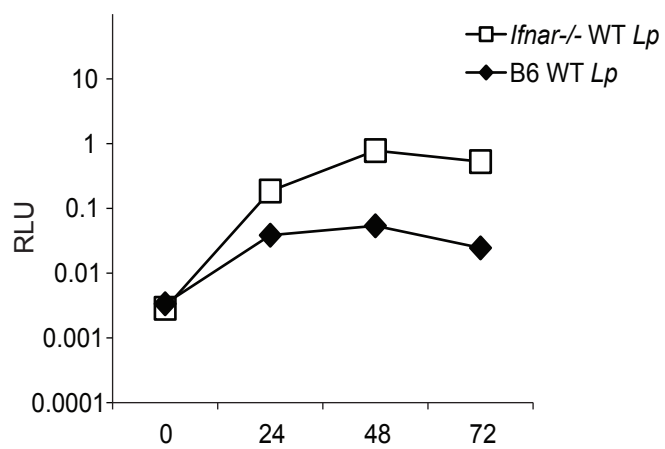

B

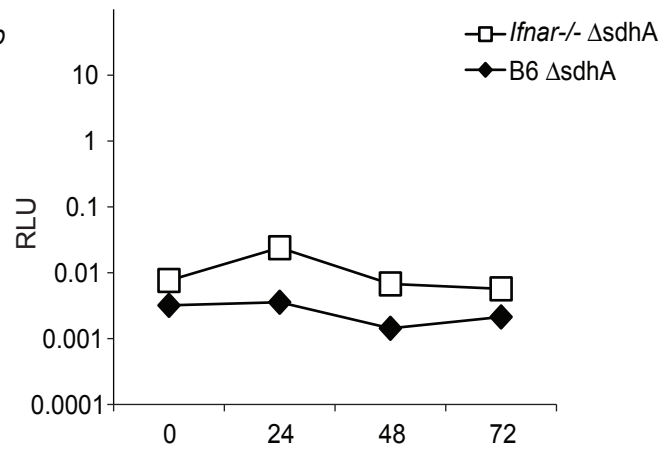

C

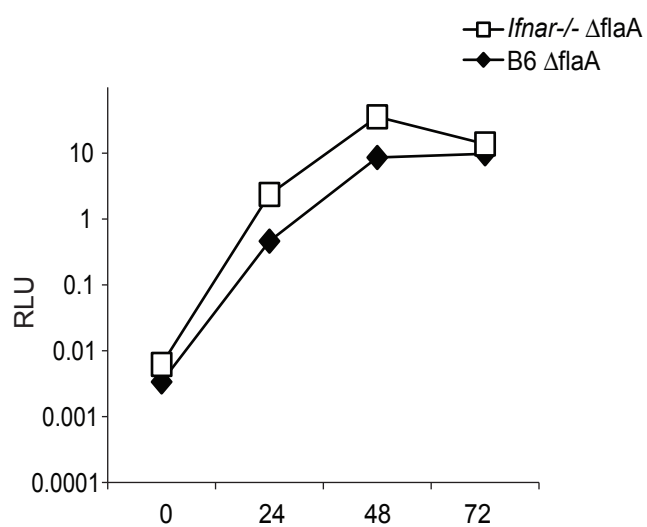

D

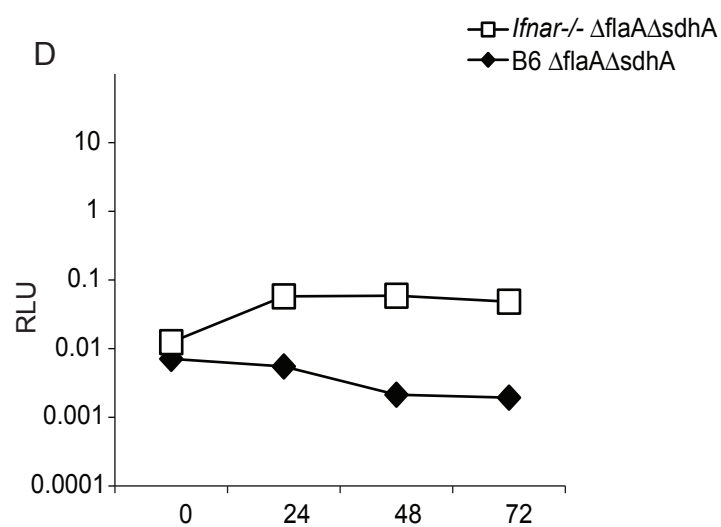

E

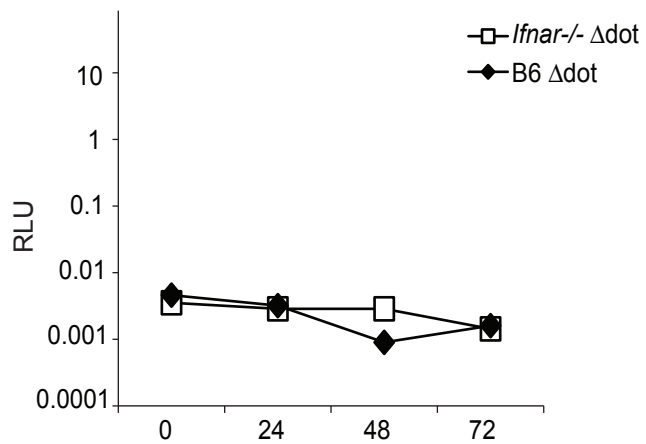

Supplement: Figure S2 — Abrogation of type I interferon receptor signaling alone does not permit growth of ΔsdhA mutant. C57BL/6 (B6) and Ifnar −/− macrophages were infected at an MOI of 0.01 and growth of luminescent L. pneumophila strains was determined by RLU at 0, 24, 48, and 72 hours post infection. (A) C57BL/6 (B6) and Ifnar −/− macrophages were infected WT (LP02) L. pneumophila (B) macrophages were infected as in A but with ΔsdhA L. pneumophila (C) ΔflaA L. pneumophila (D) ΔflaAΔsdhA L. pneumophila (E) Δdot L. pneumophila. (0.29 MB PDF) [file ppat.1000665.s002.pdf]

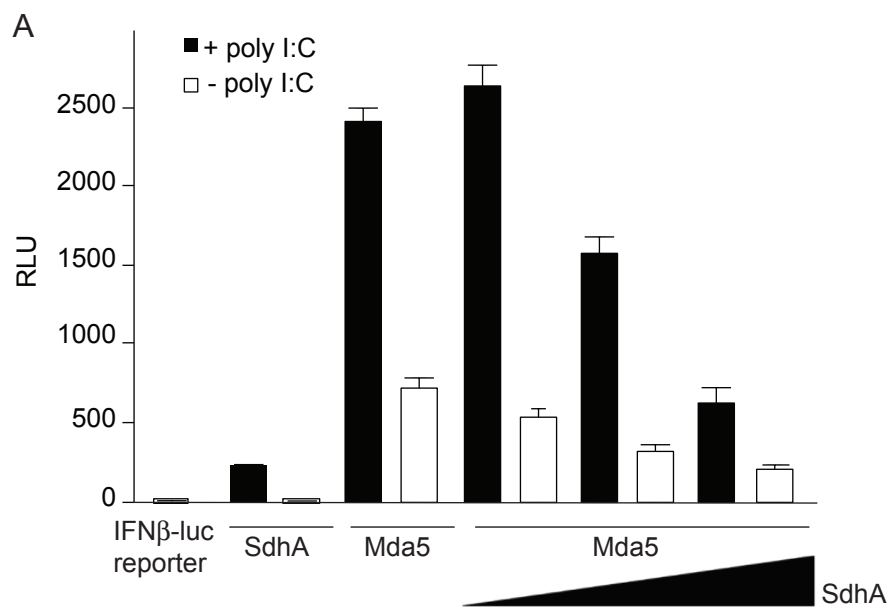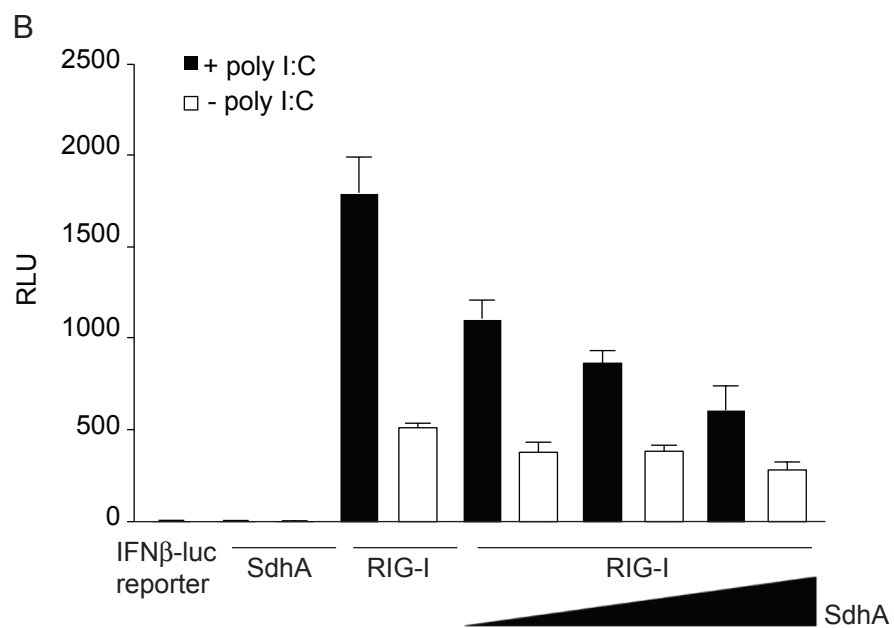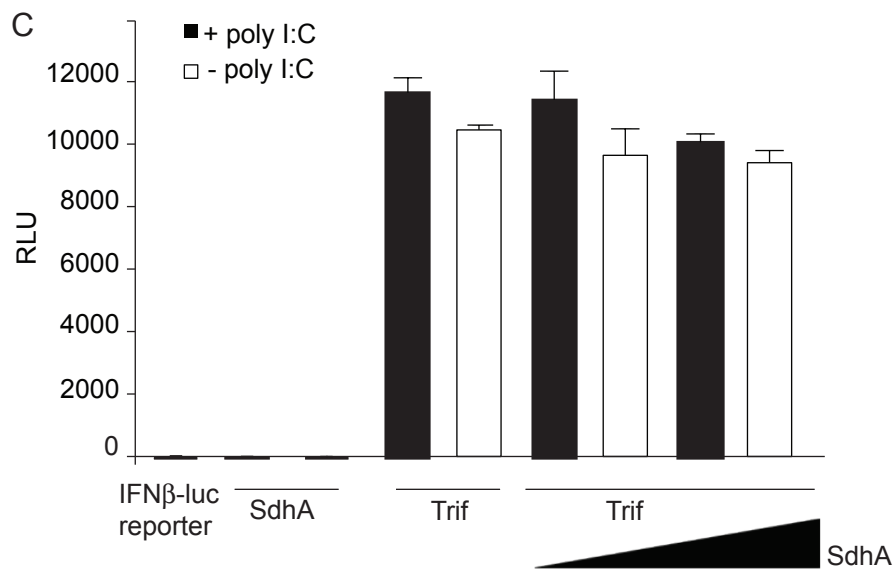

Supplement: Figure S3 — SdhA represses MDA5 and RIG-I induction of interferon. Overexpression of SdhA in HEK293T cells results in repression of interferon induction mediated by MDA5 or RIG-I but not TRIF. (A) HEK293T cells were transfected with plasmids encoding the IFNβ-firefly luciferase reporter, TK-Renilla luciferase reporter (for normalization), full length MDA5 and/or increasing amounts of full length SdhA. At 20 hours post transfection, cells were transfected with poly I:C and then firefly luciferase and Renilla luciferase levels were determined 8 hours later. (B) Transfection and stimulation were performed as in A, except with a RIG-I expression plasmid and/or increasing amounts of full length SdhA expression plasmid. (C) Transfection and stimulation were performed as in A, except with a Trif expression plasmid and/or SdhA. (0.27 MB PDF) [file ppat.1000665.s003.pdf]
